# The fatty acid imbalance of cystic fibrosis exists at birth independent of feeding in pig and ferret models

**DOI:** 10.1042/CS20220450

**Published:** 2022-12-13

**Authors:** Aliye Uc, Birgitta Strandvik, Jianrong Yao, Xiaoming Liu, Yaling Yi, Xingshen Sun, Ruth Welti, John F. Engelhardt, Andrew W. Norris

**Affiliations:** 1 Department of Pediatrics, University of Iowa, Iowa City, IA 52242, U.S.A.; 2 Fraternal Order of Eagles Diabetes Research Center, University of Iowa, Iowa City, IA 52242, U.S.A.; 3 Department of Biosciences and Nutrition, Karolinska Institutet NEO, Flemingsberg, Stockholm 14183, Sweden; 4 Department of Anatomy and Cell Biology, University of Iowa, Iowa City, IA 52242, U.S.A.; 5 Kansas Lipidomics Research Center, Kansas State University, Manhattan, KS 66506, U.S.A.

**Keywords:** cystic fibrosis, fatty acids, gastrointestinal physiology, model organisms, polyunsaturated fatty acids

## Abstract

Persons with cystic fibrosis (CF) exhibit a unique alteration of fatty acid composition, marked especially among polyunsaturates by relative deficiency of linoleic acid and excess of Mead acid. Relative deficiency of docosahexaenoic acid is variably found. However, the initial development of these abnormalities is not understood. We examined fatty acid composition in young CF ferrets and pigs, finding abnormalities from the day of birth onward including relative deficiency of linoleic acid in both species. Fatty acid composition abnormalities were present in both liver and serum phospholipids of newborn CF piglets even prior to feeding, including reduced linoleic acid and increased Mead acid. Serum fatty acid composition evolved over the first weeks of life in both non-CF and CF ferrets, though differences between CF and non-CF persisted. Although red blood cell phospholipid fatty acid composition was normal in newborn animals, it became perturbed in juvenile CF ferrets including relative deficiencies of linoleic and docosahexaenoic acids and excess of Mead acid. In summary, fatty acid composition abnormalities in CF pigs and ferrets exist from a young age including at birth independent of feeding and overlap extensively with the abnormalities found in humans with CF. That the abnormalities exist prior to feeding implies that dietary measures alone will not address the mechanisms of imbalance.

## Introduction

Cystic fibrosis (CF) is a multiorgan disease caused by genetic impairment of CF transmembrane conductance regulator (CFTR) function. Organs prominently affected include the lung, pancreas, intestine, and liver. Individuals with CF exhibit a unique disturbance of polyunsaturated fatty acid (PUFA) composition, most prominently diminished linoleic acid (18:2n6) and elevated Mead acid (20:3n9) [1–3], and often but not always low docosahexaenoic acid (22:6n3) [4–6]. The fatty acid imbalance is widespread, including in serum, liver, lung, airway, gastrointestinal tract, red blood cells (RBCs), and adipose tissue [7–10]. Likewise, the fatty acid abnormalities are present in cultured CF cells [11–14], suggesting a cell autonomous process. The biochemical mechanisms responsible for the imbalance are not fully understood [15] but include increased phospholipase activity [16–19] and elevated desaturase and elongase activities [20]. Small molecule modulators that restore CFTR function not only improve the clinical status of CF patients with responsive mutations but also influence fatty acid imbalance and levels of various complex lipids in humans with CF [4,21] and in cultured CF cells [14,22].

It is postulated that the fatty acid imbalance may contribute to CF clinical disease [23,24] and may further impair CFTR function [25]. However, the developmental relation between the appearance of fatty acid imbalance and CF tissue pathology is not well studied. The age at which CF clinical disease typically debuts varies between organs and by mutation type. Intestinal disease often has fetal or infantile onset, more so than clinically evident lung disease. Intestinal manifestations of CF often include meconium ileus in newborns, and distal intestinal obstruction syndrome and small intestinal bacterial overgrowth in adults. Exocrine pancreatic disease, evidenced by elevated serum immunoreactive trypsinogen, is often present at birth, and serves as a common approach contributing to newborn screening for CF [26]. Clinically significant pancreatic insufficiency develops during infancy in the majority of patients [27,28]. The fat malabsorption associated with CF pancreatic insufficiency is improved but not reliably eliminated by pancreatic enzyme replacement therapy [29]. However, fatty acid composition abnormalities are not ameliorated by pancreatic enzyme replacement therapy [1,7,30]. Furthermore, even CF patients who are pancreatic sufficient exhibit fatty acid composition abnormalities [10,31] .

Fatty acid composition has been studied extensively in mouse models of CF but with mixed results, depending in part on the strain and *Cftr* mutation under study [32]. Linoleic acid concentrations in CF mice have been reported to be low [11,20,32,33] or normal [34,35]. Yet several mouse studies focused on docosahexaenoic acid and arachidonic acid (20:4n6) did not report linoleic acid levels [36–38]. Age has a broad impact on fatty acid composition in CF mice, especially early in life [32]. Interestingly, one study found that low linoleic acid in mouse adipose tissue normalized at 6 weeks [39]. Diet also impacts fatty acid composition in mice [32,34]. However, there are several reasons that mice may not be an optimal model of lipid imbalance in CF disease. For one, much of human CF disease is only minimally recapitulated in the CF mouse, potentially owing to compensatory chloride currents [40]. Furthermore, docosahexaenoic acid metabolism differs between rodents and humans, with the synthesis rate being much less in humans [41,42]. Although several cell lines lacking CFTR function exhibit similar fatty acid composition abnormalities to that *in vivo* in humans with CF, this approach can not model the interplay between whole-body lipid abnormalities and organ pathology in CF.

Recently, CF models have been developed in two non-rodent species that more fully reflect human disease. These are *CFTR*-knockout and *CFTR*-ΔF508 pigs and the *CFTR*-knockout ferret [43–45]. These animal models develop severe lung, pancreatic, gastrointestinal, liver, and metabolic disease which closely resemble CF human pathology [46,47]. Gastrointestinal manifestations of CF in these models include meconium ileus, intestinal obstruction, intestinal mucoid luminal ‘plugs’, impaired bile secretion, pancreatic ductal plugging, exocrine pancreatic insufficiency, acidic pancreatic secretions and malabsorption [43–45,48]. These animal models afford new opportunities to better study the fatty acid abnormalities that accompany CF. Herein, we examined whether these CF animal models develop fatty acid composition abnormalities. We studied liver—an important hub of PUFA metabolism [49], serum/plasma, and RBCs—a marker of overall PUFA status [50]). Of particular interest was the study of the earliest abnormalities in fatty acid composition. Because fatty acid composition abnormalities have been demonstrated in CF cell cultures, this raises the possibility of cell autonomous CF effects which might induce lipid disturbance *in utero*, even before feeding and the transition to extrauterine life. As one approach to understand CF PUFA perturbations in early life, we studied fatty acid composition in RBCs, which at birth are correlated with maternal fatty acid status [51]. Additionally, we examined newborn piglets prior to any feeding, to fully isolate the impact of dietary lipid absorption from any lipid composition abnormalities that might exist at birth.

## Methods

### Animals

Gravid sows carrying CF (*CFTR*-^/^- or *CFTR*^ΔF508/ ΔF508^) and littermate non-CF (*CFTR*^+/+^, *CFTR*^+/^-, or *CFTR*^+/ ΔF508^) piglets were obtained from Exemplar Genetics (Sioux Center, IA, U.S.A.). Piglets were birthed vaginally or via caesarean delivery. Because of the extreme difficulties in rearing CF pigs past the first 24 h of life, no samples were obtained from CF pigs older than the first day of life. Several pigs without CF were raised beyond a day of life but are not included in the analyses comparing CF to non-CF animals. Some of the piglets were studied in the never-fed state whereas others were allowed to nurse. Ferrets of CF (*CFTR*^−/−^) and non-CF (*CFTR*^+/−^ and *CFTR*^+/+^) genotype were obtained from Marshall Labs (North Rose, NY, U.S.A.) and studied at ages 0–112 days. Ferrets were nursed starting at birth. For survival, CF ferrets required specialized dietary care beginning with EleCare elemental formula (Abbott, Abbott Park, Ill, U.S.A.) supplemented with pancreatic enzymes starting on day 1 of life [44]. Then at weaning, CF ferrets received EleCare plus canned food supplemented with pancreatic enzymes. These specialized diets are refused by healthy ferrets, who instead received Marshall Premium ferret chow upon weaning. Blood was drawn immediately after anesthesia with ketamine/xylazine or immediately after euthanasia with overdose of Euthasol. Sampled liver was obtained immediately after euthanasia, frozen in liquid nitrogen, and stored at −80°C.

### Analyses of fatty acid composition in various lipid fractions

Fatty acid composition was measured in three laboratories and expressed in mole%. Samples were blinded at analyses but not jointly measured between laboratories. The laboratory of measurement was codified by geographical location: Sweden, Kansas, or Iowa. Measurements were made on liver, plasma/serum, or RBC samples. In some cases, fatty acid composition was measured in whole lipid extracted from the samples. In other cases, fatty acid composition was measured in isolated lipid classes: cholesterol esters, lysophospholipids, phospholipids, sphingomyelins, or non-esterified fatty acids. Methods for each laboratory are detailed in the following paragraphs.

#### Porcine serum and liver fatty acid composition

These measurements were performed in the research laboratory of BS in Sweden. For plasma samples, fatty acids were analyzed after lipid extraction with addition of two drops of butylated hydroxytoluene (BHT) (0.1 mg/ml chloroform) [52] and isolation of phospholipid fraction by SEP-PAK aminopropyl cartridge (Waters Corp., Milford, MA, U.S.A.) eluted with methanol after washing with chloroform:isopropanol 2:1 and 2% acetic acid in ether. The resulting phospholipid fraction was transmethylated in methanolic-HCl-3N at 80°C over 4 h. The fatty acid methyl esters (FAME) were extracted with hexane, washed with water, dried over MgSO_4_, and dissolved in hexane All evaporation steps were performed under nitrogen. The FAME were separated by capillary gas chromatograph in a Hewlett-Packard 6890 gas chromatograph (HP, Wilmington, DE, U.S.A.) equipped with a 30 m × 0.25 mm 20 μm film thickness SP-2380 column. Helium at 1.4 ml/min was used as carrier gas. The injector and detector temperatures were 250°C. The column oven temperature was programmed from 60 to 230°C at a heating rate of 8°C/min up to 155°C, 1.5°C/min up to 180°C and thereafter 6°C/min up to 230°C, where it was run for 10 min. Eluting FAME were detected by flame ionization. The separation was recorded with HP Chem Station software. Heneicosanoic acid (21:0) was used as an internal standard and the FAME identified by comparison with retention times of pure reference substances (Sigma-Aldrich Sweden AB, Stockholm, Sweden).

While still chilled, freshly thawed liver samples were weighed, minced into pieces and homogenized in water with a Polytron PT 1200 CL (Kinematica AG). Lipids were extracted using chloroform and 2-propanol (7:11 v/v) with BHT and sonication [53]. The phospholipid fractionation high-performance liquid chromatography method [54] was slightly modified for use of an internal standard and to collect lipid fractions from split post column flow. The system consisted of two delivery pumps (Bischoff 2250), an injector of 20 µl, a gradient mixing chamber 1.8 mL (SPARK) and a detector ELSD Varex MKIII (Alltech). The column was a LiCrospher 100 Diol 5 µm 250 × 4 mm with a Si guard column. The column temperature was 55°C. Pump control and detector signal evaluation were managed by Clarity (DataApex LTD, Prague) software. Standards were composed of mixtures of phospholipids from Larodan Fine Chemicals (Malmö, Sweden). Fractions corresponding to phosphatidylcholine, phosphatidylethanolamine, phosphatidylserine, phosphatidylinositol, and sphingomyelins were collected. The fractions were dried under nitrogen, and FAME prepared and analyzed by capillary gas chromatography as described above.

#### Mass spectrometry of pig and ferret plasma lipids

These measurements were made at the Kansas Lipidomics Research Center at Kansas State University, Manhattan, Kansas. Plasma (3 μl) was added directly to mass spectrometry solvent. Samples were prepared and phospholipids and cholesterol esters were analyzed by direct-infusion electrospray ionization triple quadrupole mass spectrometry, as described [55]. Non-esterified fatty acid composition was determined by scanning with a single quadrupole in negative ion mode.

#### Fatty acid composition in whole lipid extract from porcine and ferret RBC and ferret liver:

These measurements were made at the University of Iowa, Iowa City, Iowa. RBCs were isolated from fresh blood samples and stored at −80°C before lipid extraction. Lipid was extracted from RBCs using chloroform-isopropanol as described [53] using isopropanol containing 0.005% BHT. Lipid was extracted from homogenized liver using chloroform-methanol and saline wash [52]. The chloroform-methanol mixture contained 0.005% BHT. For both extraction methods, the organic solvent of the resulting lipid fractions was evaporated under nitrogen. Dried lipids were methyl transesterified using methanol-hexane-acetyl chloride [56] containing 0.0025% BHT under nitrogen. The resulting non-polar fraction was recovered by addition of heptane and then evaporated under nitrogen. The resulting dried FAME were reconstituted into heptane for injection onto a DB23 capillary column (30 m length × 0.25 mm inner diameter × 0.25 μm film, Agilent, Santa Clara, CA, U.S.A.) housed in a HP5890 gas chromatograph. The sample injection volume was 1 microliter. The split ratio was 200:1. The carrier gas was helium at constant column head pressure of 83 kilopascals. The column oven temperature was 50°C for 1 min, 5 min ramp to 175°C, 17 min ramp to 225°C, then 230°C for 56 min. Effluent contents were detected by flame ionization. In addition to the usual cis-fatty acids, several trans-fatty acids were resolved by this method. Peaks were identified using four standards: NuChek 68B (Elysian, MN), Sigma 37 component FAME mixture, Sigma PUFA1 mixture, and Sigma PUFA2 mixture (Millipore Sigma, St. Louis, MO, U.S.A.).

### RT-PCR

Hepatic RNA was treated with the DNase (pig: DNase1 kit, New England BioLabs, Ipswich MA, U.S.A.; ferret: Turbo DNase, Ambion, Austin TX, U.S.A.). One microgram of RNA was used to generate first-strand cDNA (pig: SuperScript II, Invitrogen, Carlsbad CA, U.S.A.; ferret: Kit #4368814, Applied Biosystems, Carlsbad CA, U.S.A.). Sequences for primers (Integrated DNA Technologies, Coralville IA, U.S.A.) are shown in Supplementary Table S1. Real-time PCR reactions utilized Power SYBR Green PCR Master Mix (Applied Biosystems). RNA amount was calculated relative to the indicated reference transcript(s) using the 2^−ΔΔCT^ method.

### Statistics

Unless otherwise stated, data are presented as the mean ± standard error of measurement. Statistical tests were performed in R (version 3.3.3, R Foundation for Statistical Computing, Vienna, Austria), including principal component analysis. Significance under conditions of multiple hypothesis testing was assessed through calculation of the false discovery rate (FDR) [57]. Normality was assessed by the Shapiro–Wilk test.

#### Unified dataset

Where specified, analyses were performed on a unified dataset, integrating the composition data for 27 specific fatty acids among the three laboratories (Supplementary Table S2). For saturated fatty acids, this integration was fully straightforward. For unsaturated fatty acids measured by mass spectrometry, the unsaturation positioning was unknown. For several fatty acids this precluded unambiguous integration, and thus these measurements were omitted from the unified dataset (5 fatty acids, Supplementary Table S3). Fatty acids of very minimal relative abundance (<0.1%) measured only in one laboratory were also omitted from the unified dataset (6 fatty acids), as were fatty acids whose peaks were not resolved by gas chromatography (1 fatty acid, Supplementary Table S3).

#### Factor analysis on mixed data

Factor analysis on mixed data was performed using the *FactoMineR* package [58] in R on the unified dataset. The data matrix was necessarily incomplete because not all fatty acids were measured in all laboratories. Empty values in the matrix were imputed using the *missMDA* [59] package in R, for input along with the raw matrix into the *FactoMineR* algorithm using default parameters.

#### Linear modeling

Linear modeling was performed in R, calculating p-values using type-III analysis-of-variance tables as implemented in the *car* [60] R package. For datasets containing random factors, linear mixed effects modeling was performed using the *lme4* [61] package in R. Animal age was classified into the following levels: 0–24 h old ‘newborn’, 1–42 days old ‘pre-weanling’, 43–150 days old ‘juvenile’, and 151 days and older ‘adult’.

### Study approval

All studies involving live animals were performed at the University of Iowa and were approved by the University of Iowa Animal Care and Use Committee.

## Results

### Impact of genotype, relative to other factors, on fatty acid composition

**Figure 1:**
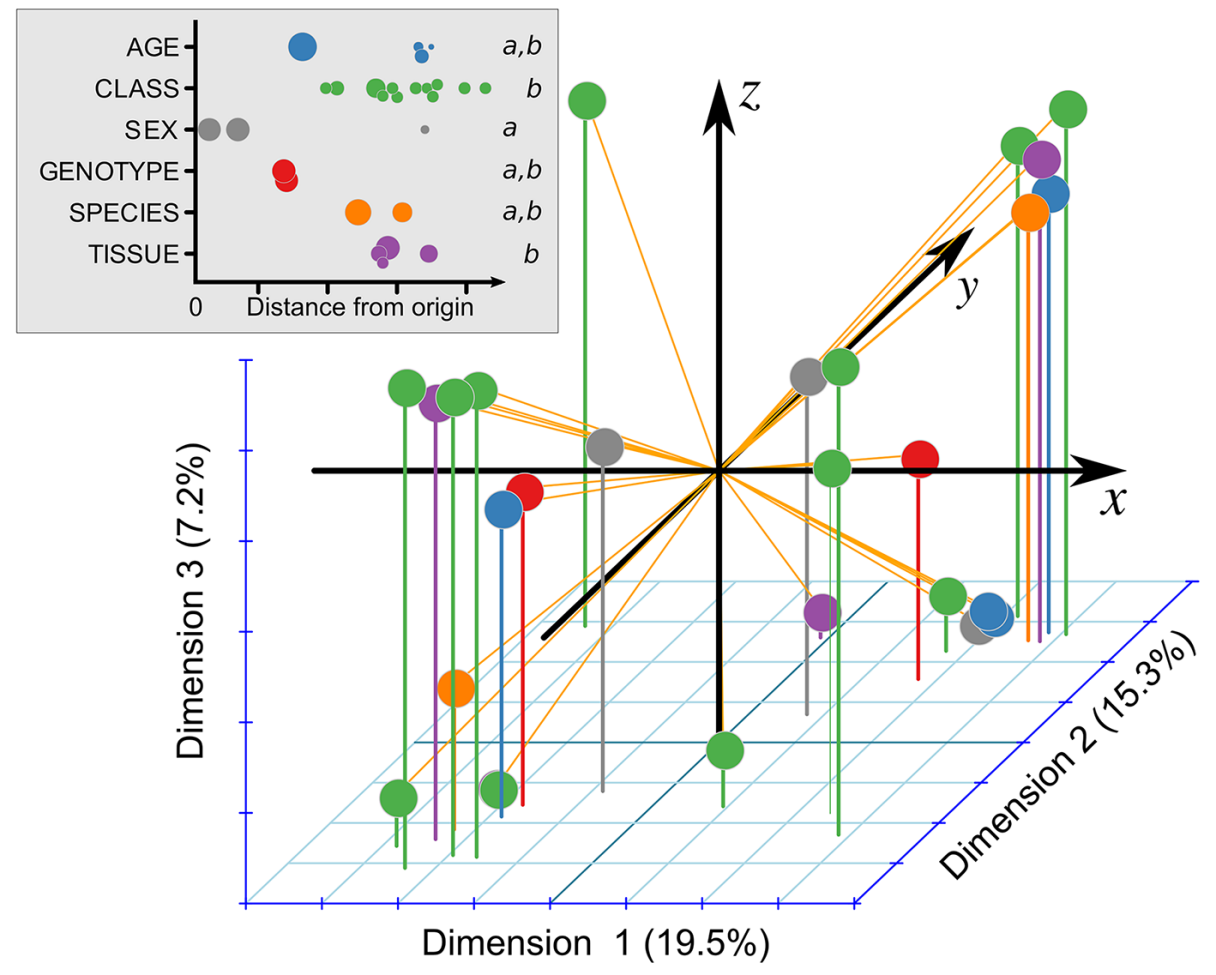
Relative influence of genotype (CF versus non-CF) and other factors on overall fatty acid composition Composition of 27 major fatty acids was subject to factor analysis on mixed data. The resulting three principal dimensions in log_10_ space are shown, with the explained variance per axis noted in parentheses. The upper-left inset shows the colors for each of the factors, as well as the distance from the origin for each point. The size of each point is proportional to the numbers of samples represented by the point. Italicized ‘*a*’ and ‘*b*’ indicate statistical groupings as determined by weighted one-way ANOVA followed by Tukey’s HSD.

We measured mole percent fatty acid composition in samples collected from young CF pigs and CF ferrets, using their non-CF counterparts as controls. The full dataset, available online (https://doi.org/10.7910/DVN/5NB36Y), is overviewed in Table 1. Samples were collected at various ages (range: 0.5–337 days), with 85% of samples collected before 2 days of age. Only 1 pig and 1 ferret underwent repeated sampling, once for each. Samples were obtained from liver (54%), plasma/serum (29%), or RBCs (17%). Most analyses included fractionation steps to allow determination of the fatty acid composition of specific lipid classes, 7% from cholesterol esters, 19% lysophospholipids, 28% phospholipids, 12% sphingomyelins, 4% non-esterified fatty acids, whereas 30% examined fatty acid composition in the general lipid fraction. Samples were collected from both sexes, 51% from men. Some samples were collected from newborn animals that had never been fed (62%). To better understand the impact of each of these variables on fatty acid composition, we performed factor analysis on mixed data (Figure 1). This demonstrated that sex (gray circles) contributed less to the variation in fatty acid composition than lipid class and tissue of study. Other variables also impacted fatty acid composition, including genotype, species, and age (Figure 1).

**Table 1:** Overview of entire data set, showing *n* for various data components

*n*	Total	Pig	Ferret
Animals	165	81	84
Samples^*^	517	351	166
Measurements	11278	7908	3370

^*^For the purpose of this table, the individual lipid classes fractionated from biological specimens are considered as separate samples.

### Individual comparisons of fatty acids between CF and non-CF, in each lipid class and tissue

**Figure 2:**
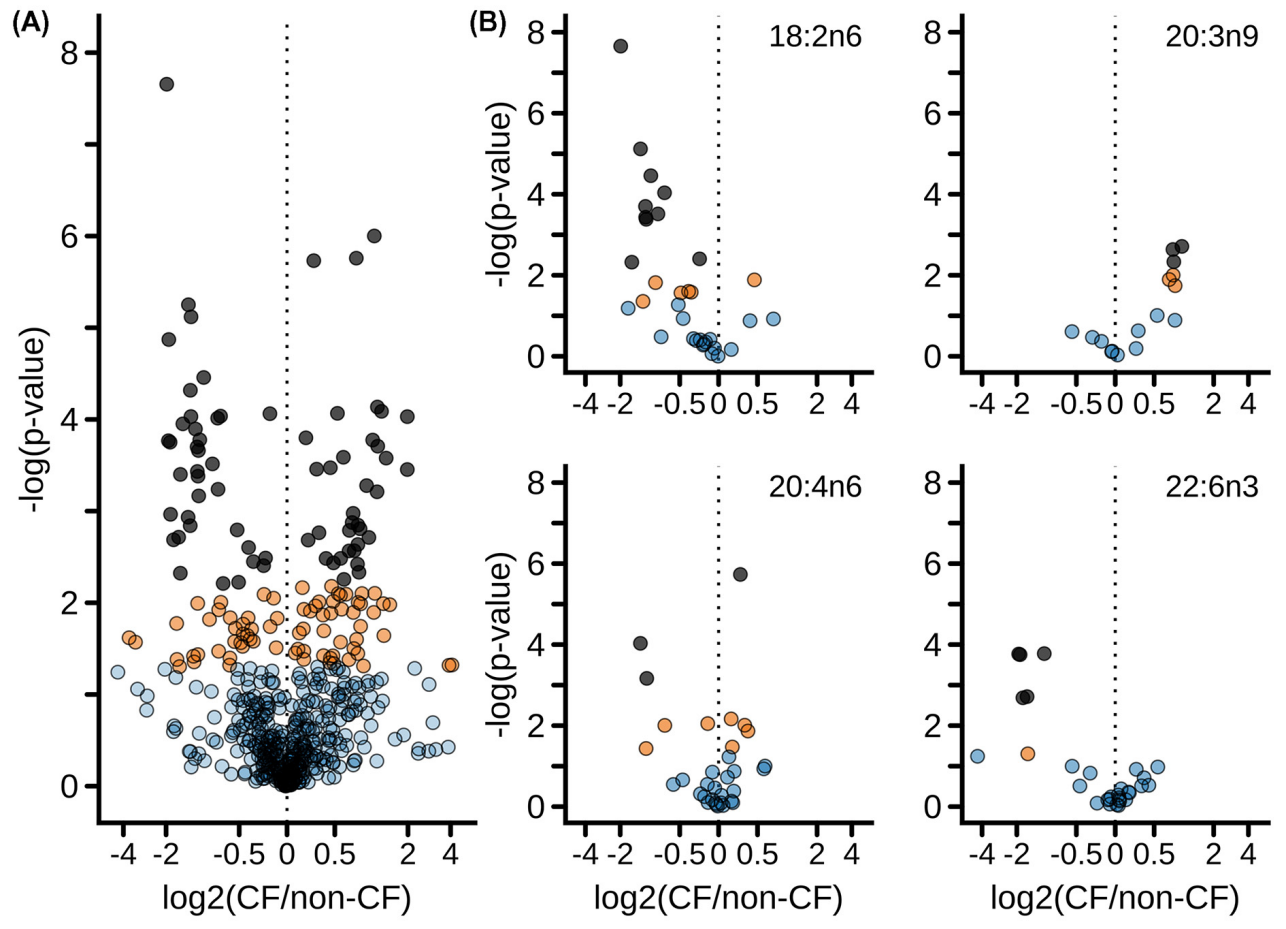
The impact of CF on fatty acid composition (**A**) Volcano plot showing changes in composition of individual fatty acids between CF and non-CF samples, isolating the non-genotype factors of species, tissue examined, lipid class, fed and fasting status, birth method, and age category. Each dot represents a specific fatty acid as measured in a specific species, tissue, lipid class, etc. Comparisons reaching a false discovery rate (FDR) of <0.05 are shown in dark grey fill. Comparisons reaching nominal significance with *P*<0.05 but false discovery rate > 0.05 are shown in orange fill. Comparisons not reaching significance (i.e. *P*>0.05) are shown with blue fill. (**B**) Volcano plot of selected fatty acids as indicated.

The dataset allowed 559 distinct pairwise comparisons of fatty acid composition between CF and non-CF samples, isolating other important variables including species, lipid class, feeding status, age, and tissue. These 559 comparisons are represented as a volcano plot (Figure 2A). Of the 559 pairwise comparisons, 70 achieved stringent significance of FDR<0.05 (Figure 2A dark gray symbols; details are tabulated in Supplementary Table S4, sorted by fatty acid and by species), whereas 75 exhibited nominal significance with *P*<0.05 but FDR>0.05 (Figure 2A, orange symbols). Table 2 lists the 13 which met the strict Bonferroni criteria of *P*<0.00009. The list of all 559 comparisons is available online (https://doi.org/10.7910/DVN/5NB36Y). The portions of the volcano pertaining to linoleic acid (18:2n6), Mead acid (20:3n9), arachidonic acid (20:4n6), or docosahexaenoic acid (22:6n3) are shown in Figure 2B. These show that linoleic acid and docosahexaenoic acid compositions were significantly diminished by CF under a variety of conditions, whereas that of Mead acid was significantly increased under some conditions. By contrast, significant changes in arachidonic acid composition were increased or decreased depending on condition.

**Table 2:** Fatty acid composition strongly impacted by CF

Fatty acid	Tissue	Class	Mole%		Age	Species	*N*	*P*-value
			Non-CF	CF				
16:0	Liver	L	20.7 ± 0.4	18.5 ± 0.3	Newborn	Pig	7.13	9E−05
16:1n7	Liver	PC	4.0 ± 0.3	5.7 ± 0.2	Newborn	Pig	7.13	9E−05
18:1*	Plasma	LPE	20.4 ± 3.5	49.5 ± 1.8	Newborn	Ferret	5.5	8E−05
18:2n6	Liver	PC	9.4 ± 1.2	4.6 ± 0.2	Newborn	Pig	7.13	3E−05
	Liver	PE	4.7 ± 0.6	1.9 ± 0.1	Newborn	Pig	7.13	8E−06
	Serum	PL	15.9 ± 1.6	4.0 ± 0.4	Newborn	Pig	7.13	2E−08
20:1*	Plasma	NEFA	0.6 ± 0.1	1.4 ± 0.1	Newborn	Ferret	5.5	7E−05
20:2n6	Liver	PC	0.2 ± 0.0	0.1 ± 0.0	Newborn	Pig	7.13	5E−05
	Liver	PE	0.2 ± 0.0	0.1 ± 0.0	Newborn	Pig	7.13	1E−05
	Serum	PL	0.2 ± 0.0	0.1 ± 0.0	Newborn	Pig	7.13	6E−06
20:3n6	RBC	L	0.4 ± 0.0	0.9 ± 0.0	Juvenile	Ferret	6.5	1E−06
20:4n6	Liver	PE	24.4 ± 0.7	29.3 ± 0.4	Newborn	Pig	7.13	2E−06
24:1n9	RBC	L	1.2 ± 0.0	2.1 ± 0.1	Juvenile	Ferret	6.5	2E−06

*Unsaturated fatty acids without an omega indicator were measured by mass spectrometry and thus positional information was not determined. Species 18:1 is a mixture of different omega isomers, typically mostly 18:1n9 but containing varying relative amounts of 18:1n7. By contrast, species 20:1 is nearly exclusively isomer 20:1n9.

Of 559 pairwise CF versus non-CF comparisons, 13 met statistical significance accounting for multiple hypothesis testing, with *P*<0.00009. Lipid classes: L, total lipid; LPE, lysophosphatidylethanolamine; NEFA, non-esterified fatty acids; PC, phosphatidylcholine; PE, phosphatidylethanolamine; PL, all phospholipids. *N* is the number of non-CF and CF animals examined.

### Overall impact of CF on fatty acid composition in newborns

**Figure 3:**
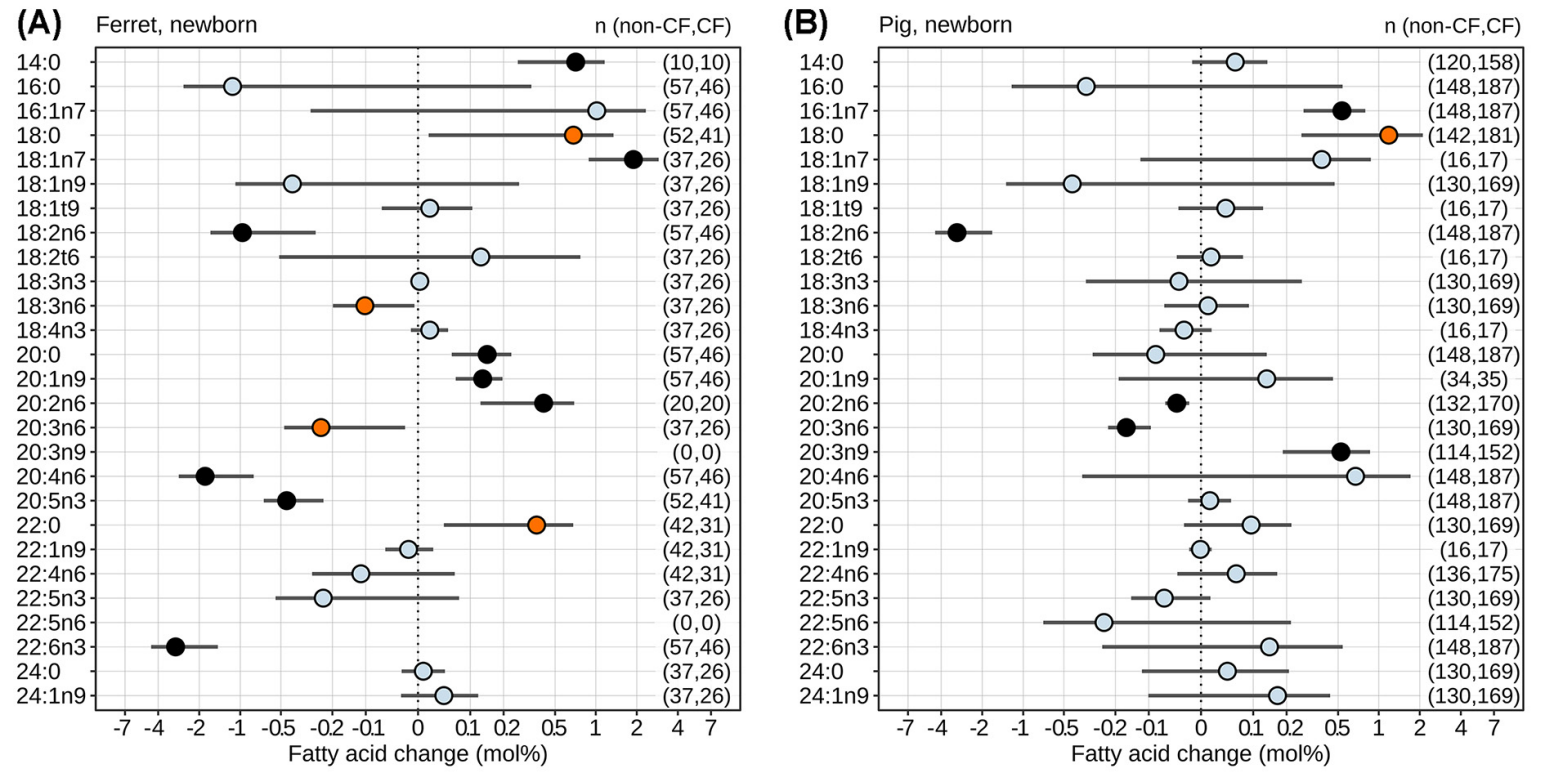
The impact of CF on fatty acid composition in newborns The effect of CF in (**A**) ferrets and (**B**) pigs on fatty acid mole% was determined by linear mixed effects modeling across the unified dataset, accounting for multiple co-variates. Mean effects (CF minus non-CF) and 95% confidence intervals (points and bars) are shown and account for multiple co-variates as described in Methods. The following factors were treated as fixed effects: CF versus non-CF, lipid class examined, tissue examined, method of birth (C-section versus vaginal), sex, never fed status, diet, and length of fast before sample collection. Animal subject and lab of analysis were treated as random factors. The two species were analyzed separately. Equivalent fatty acids from the three differing methodologies were analyzed jointly. The statistical significance of difference for each fatty acid is indicated by color: light blue, non-significant (*P*>0.05); orange, *P*<0.05 but false discovery rate (FDR) > 0.05; black, FDR < 0.05. The number of measurements (*n*) underlying each fatty acid assessment are shown in the right-hand side (non-CF, CF).

To assess the overall association between CF and fatty acid composition across the entire dataset and the multiple conditions studied, we performed linear mixed effects modeling on data collected from newborn animals, isolating the impact of CF versus non-CF genotype while accounting for important covariates. The pattern of several fatty acids was significantly perturbed in newborns by CF (Figure 3). Among PUFAs, there was a significant decrease in linoleic acid (18:2n6) in both newborn ferrets (Figure 3A) and newborn pigs (Figure 3B). Mead acid (20:3n9) was increased in newborn pigs, but was not resolved in newborn ferrets. Several PUFAs were only diminished in newborn ferrets, namely docosahexaenoic acid (22:6n3), arachidonic acid (20:4n6), and eicosapentaenoic acid (20:5n3). Dihomo-γ-linolenic acid (20:3n6) was significantly diminished in newborn CF pigs and CF ferrets, though the latter only reached nominal significance. Eicosadienoic acid (20:2n6) was significantly increased in newborn CF ferrets whereas it was diminished in newborn CF pigs. The relative abundances of several saturated and monounsaturated fatty acids were increased by CF, including myristic acid (14:0), vaccenic acid (18:1n7), arachidic acid (20:0), and gondoic acid (20:1n9) in newborn ferrets, and palmitoleic acid (16:1n7) in newborn pigs, but none were decreased.

### Fatty acid composition in hepatic and serum/plasma lipids prior to initial feeding after birth

**Figure 4:**
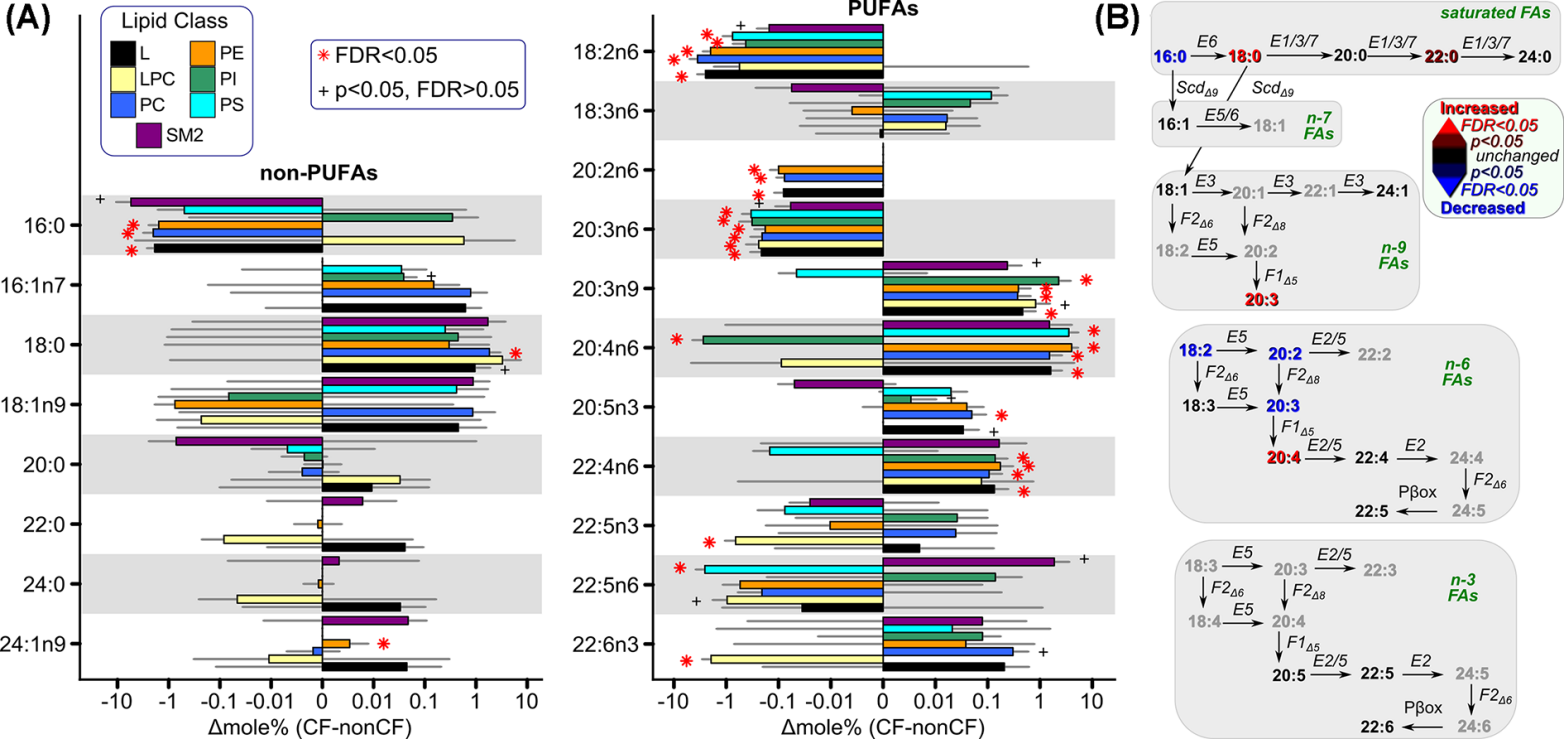
Neonatal, pre-feeding defects in fatty acid composition Alterations of fatty acid mole% in liver lipids of never-fed CF piglets compared with non-CF controls. (**A**) Mole% differences between CF and non-CF liver as measured in specific lipid classes. Significance was assessed by linear modeling with FDR<0.05 indicated by red asterisk; nominal significance (*P*<0.05; FDR>0.05) indicated by plus sign; *N* = 11–17. The *X*-axis is linear from −0.01 to 0.1 and logarithmic otherwise. Lipid classes are: L, whole lipid extract; LPC, lysophosphatidylcholine; PC, phosphatidylcholine; PE, phosphorylethanolamine; PI, phosphatidylinositol; PS, phosphatidylserine; SM2, sphingomyelin 2. (**B**) Overall changes in fatty acid composition in never-fed piglet liver were mapped on to fatty acid synthetic pathways (uninvolved downstream portions of pathways are not shown). Significance was assessed by linear mixed effects modeling. Gray boxes house the differing unsaturation groupings. Fatty acids with increased composition are red; decreased blue; unchanged black; unmeasured fatty acids are in gray. Color brightness indicates statistical significance (bright color FDR<0.05; dark color *P*<0.05 with FDR>0.05). Rightward arrows represent elongation; downward desaturation; leftward β-oxidation. Enzymes involved are italicized: E = elongases Elovl1/2/3/5/6/7; F = desaturases Fads1/2 indicating the desaturase activity involved Δ5/6/8; Scd = stearoyl-CoA desaturase; Pβox = peroxisomal β-oxidation.

We thus sought to determine the earliest abnormalities in fatty acid composition in CF. To address this question, we measured hepatic fatty acid composition in never-fed newborn pigs. Examining fatty acid composition in specific lipid classes, we found multiple disturbances (Figure 4A). Among non-PUFAs (Figure 4A, left panel), palmitic acid (16:0) was significantly decreased in whole lipid extract, phosphatidylcholine, and phosphatidylethanolamine, whereas stearic acid (18:0) and nervonic acid (24:1n9) were increased in lysophosphatidylcholine and phosphatidylethanolamine, respectively. By contrast, PUFAs were more consistently affected by CF (Figure 4A, right panel). Significant decreases occurred in linoleic acid (18:2n6) (all lipid classes tested except lysophosphatidylcholine and sphingomyelin), eicosadienoic acid (20:2n6) (all classes tested), dihomo-γ-linolenic acid (20:3n6) (all classes tested except sphingomyelin), clupanodonic acid (22:5n3) (only among lysophosphatidylcholine), osbond acid (22:5n6) (only among phosphatidylserine), and docosahexaenoic acid (22:6n3) (only among lysophosphatidylcholine). Significant increases occurred in Mead acid (20:3n9) (whole lipid extract, phosphatidylcholine, phosphatidylethanolamine, and phosphatidylinositol), eicosapentaenoic acid (20:5n3) (phosphatidylcholine), and adrenic acid (22:4n6) (whole lipid extract, phosphatidylcholine, phosphatidylethanolamine, and phosphatidylinositol). Mixed results were observed for arachidonic acid (20:4n6), with both significant increases (whole lipid extract, phosphatidylcholine, phosphatidylethanolamine, phosphatidylserine, and sphingomyelin) and decreases (phosphatidylinositol) depending upon lipid class. Only γ-linolenic acid (18:3n6) exhibited no evidence of significant alteration. To provide a more integrated view of the alterations in fatty acid composition in never-fed pig liver, we assessed fatty acid composition across all lipid classes and mapped the results on to fatty acid synthetic pathways [42,62] (Figure 4B). Among saturated fatty acids, palmitic acid (16:0) was significantly decreased, whereas stearic acid (18:0) was increased. Downstream of these nonessential fatty acids, there was a significant increase in Mead acid (20:3n9). Among n-6 fatty acids, linoleic (18:2n6), eicosadienoic (20:2n6), and dihomo-γ-linolenic (20:3n6) were decreased whereas arachidonic (20:4n6) was increased. By contrast, none of the measured n-3 fatty acids showed evidence for global disturbance. These changes in hepatic fatty acid composition were partly reflected in the serum phospholipids and plasma lipids of never-fed CF versus non-CF piglets, in which there were significant decreases in linoleic acid (18:2n6) and increases in palmitoleic acid (16:1n7) and nervonic acid (24:1n9) (Supplementary Figure S1). There was also a nominal decrease in eicosadienoic acid (20:2n6), and nominal increases in Mead acid (20:3n9), adrenic acid (22:4n6), and lignoceric acid (24:0).

### Early life RBC fatty acid composition in CF

**Figure 5:**
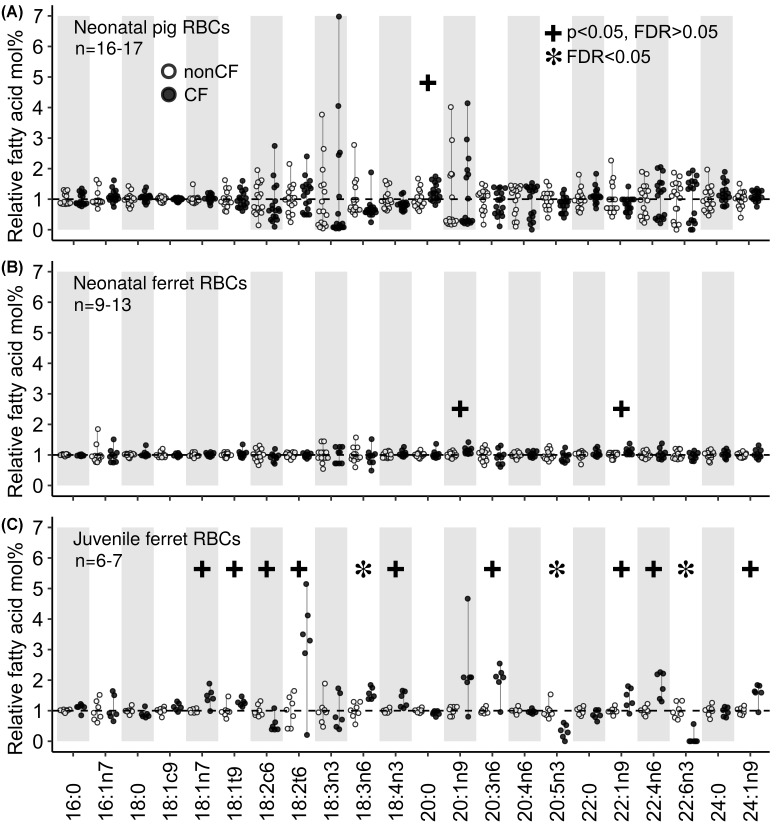
The effect of CF on RBC lipid fatty acid composition in neonatal (0–24 h of age) and juvenile (56–112 days of age, mean = 92 ± 6.0 days) animals RBCs were collected from non-CF (open circles) and CF (dark circles) (**A**) neonatal pigs, (**B**) neonatal ferrets, and (**C**) juvenile ferrets. Data are expressed as relative fatty acid mole% normalized to the mean observed in non-CF animals. Stars indicate FDR < 0.05, whereas pluses indicate *P*<0.05 but FDR > 0.05.

We examined the fatty acid composition of the phospholipid fraction from RBCs collected from newborn ferrets and pigs (Figure 5A,B). Samples were collected from birth-sibling cohorts who shared the same *in utero* environment. In newborn RBCs, there were nearly no differences between CF and non-CF fatty acid composition in either species, including no significant difference in linoleic acid (18:2n6) (*P*=0.36 and 0.43 for ferrets and pigs respectively) or docosahexaenoic acid (22:6n3) (*P*=0.57 and 0.92 for ferrets and pigs respectively). By contrast, RBC membrane phospholipid composition was markedly abnormal in juvenile CF ferrets as compared with controls (Figure 5C). The disturbances included reduced linoleic acid (*P*=0.009, FDR = 0.36) and docosahexaenoic acid (*P*=0.00003, FDR = 0.002).

### Evolution of fatty acid composition abnormalities over time in CF

**Figure 6:**
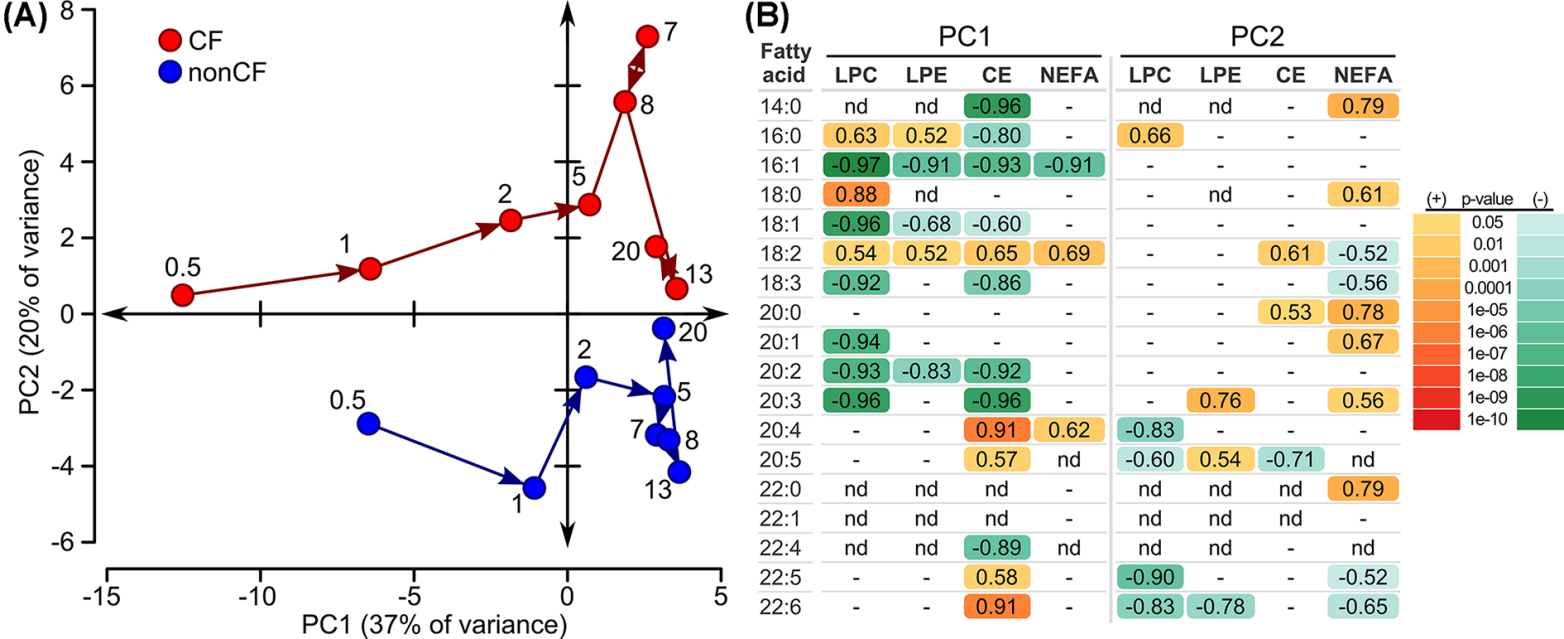
Ferret plasma lipid fatty acid composition changes over the first 3 weeks of life *N* = 11 ferrets per genotype, without repeated sampling, 1–4 samples per time point, 4 lipid classes represented (lysophosphatidylcholine LPC, lysophosphatidylethanolamine LPE, cholesterol esters CE, non-esterified fatty acids NEFA). Fifty-nine discrete lipid species were measured across these 4 classes, among 18 fatty acids. Unsaturated fatty acids were measured by mass spectrometry and thus omega isomer positional information was not determined. (**A**) Principal component analysis, plotting the changes in fatty acid profile of CF (red) versus non-CF (blue) ferrets over time. The age in days for sample collection is indicated for each point on the graph. (**B**) Lipid species significantly correlated with principal component axes 1 and 2 (PC1, PC2). Correlation values are shown. Only significant correlations (*P*<0.05) are shown, and* P*-values are coded by color, with different color scales used depending on whether the correlation was negative or positive. Lipid species that were not significantly correlated are marked as ‘-’. Lipid species that were not detected are marked ‘nd’.

Serial plasma samples were collected from CF and non-CF ferrets over the first several weeks of life, measuring fatty acid composition in cholesterol esters, lysophosphatidylcholine, lysophosphatidylethanolamine, and non-esterified fatty acids. The results were examined using principal component analysis (Figure 6A). The two leading principal components accounted for 57% of the variance in fatty acid composition. When mapped onto these principal components, data from CF versus non-CF ferrets showed interesting patterns over time. Along the axis of principal component 1 (PC1), both CF and non-CF fatty acid composition showed similar behavior. Both moved rapidly to the right after birth, to higher values of PC1, eventually reaching a relatively stable position by 5–7 days of life. Thus, PC1 largely represents changes that occurred over time in fatty acid composition and these changes were similar between genotypes. By contrast, along the axis of principal component 2 (PC2) the two groups exhibited different behavior whereby the CF animals remained in the top quadrants and the non-CF animals remained in the bottom quadrants. Thus, PC2 represents differences between CF and non-CF fatty acid composition. When considering the fatty acid composition of each lipid class separately, the same general patterns (i.e. PC1: left to right over time, PC2: CF above non-CF) mapped onto the axes (Supplementary Figure S2), with lysophosphatidylcholine and cholesterol esters contributing quantitatively to a larger degree to the PC1 changes, non-esterified fatty acids more so to PC2. The mole% over time for the top contributing fatty acids to each of the four cardinal plot directions exemplify the composition patterns that comprise the principal components (Supplementary Figure S3). To summarize these four exemplars, 16:1 in lysophosphatidylcholine and 20:4 in cholesterol esters exhibit changes over time which are similar between the two groups and exemplify the negative and positive patterns comprising PC1. In contrast, 14:0 in non-esterified fatty acids and 22:5 in lysophosphatidylcholine exhibited fewer changes over time, but exhibited stable positive and negative differences between the CF and non-CF groups respectively. All lipid species significantly correlated with PC1 and PC2 are shown in Figure 6B. Interestingly, 16:1 and 18:2 exhibited a reciprocal relationship in PC1 and were the only fatty acids that contributed across the four lipid classes. By contrast, no individual fatty acid contributed to PC2 consistently across the four lipid classes.

### Serum phospholipid composition

**Figure 7:**
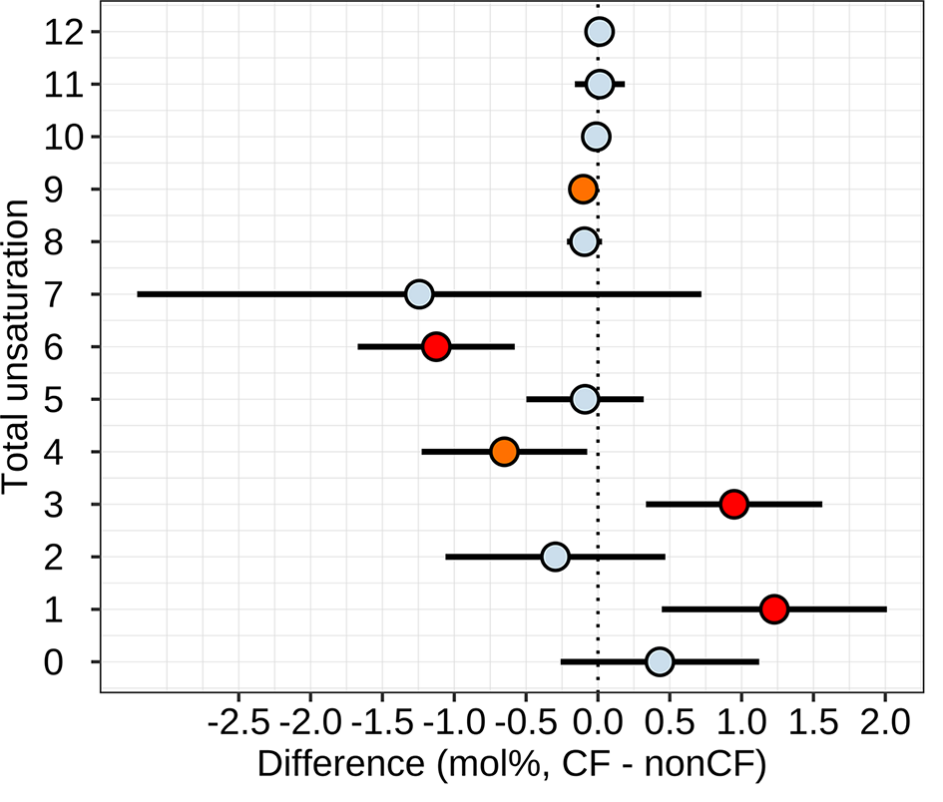
Phospholipid unsaturation as impacted by CF in ferrets Phospholipids were grouped by total unsaturation number. The impact of genotype on each groups’ mole% was examined using linear mixed effects modeling. Mean effects and 95% confidence intervals (points and bars) are shown and account for multiple covariates as described in Methods. The statistical significance of difference for each fatty acid is indicated by color: light blue, non-significant (*P*>0.05); orange, *P*<0.05; black, FDR < 0.05; read *P*<0.05/12. *N* = 11 non-CF and *N* = 11 CF independent samples.

The mass spectrometry studies yielded quantitation of phospholipid species containing two fatty acids acyl chains per molecule. Because the individual fatty acyl moieties were not resolved by this approach, these data were not included in the above fatty acid analyses, but instead are reported here. The full dataset is publicly reposited (https://doi.org/10.7910/DVN/5NB36Y); all mass spectrometry data are also reposited in concentration units. A total of 292 phospholipid species were quantified. Single serum samples were obtained from 6 CF and 5 non-CF pigs all aged 1 day. Single serum samples were obtained from 11 CF and 11 non-CF ferrets aged 0.5–20 days, using a paired approach. Phospholipids composition was impacted by age, species (pig versus ferret), genotype, and sex (Supplementary Figure S4). All 292 phospholipid species were examined for differences between CF and non-CF genotypes, using mixed effects modeling to account for the impact of sex, age, and diet, treating ferret pairings as random effects. For pig, the analysis was underpowered, such that no differences reached statistical significance when accounting for multiple hypothesis testing (Supplementary Figure S5). By contrast, for ferrets, genotype impacted 84 phospholipids evidenced by FDR<0.05 (Supplementary Figure S5) with 41 of these meeting the stringent Bonferroni criteria of *P*<0.05/292 (Supplementary Figure S6). We noticed that many of the phospholipids diminished by CF often had higher degrees of unsaturation, and those increased by CF often had lower degrees of unsaturation. Thus, we tested whether there was a consistent difference in levels of phospholipids when grouped by unsaturation degree. Indeed, in the ferret samples, phospholipids with 6 or 9 total double bonds were diminished by CF, whereas those with 1 or 3 double bonds were increased (Figure 7). The same analysis performed on the pig data yielded no results reaching statistical significance. Phospholipid composition in ferrets evolved over time with a pattern like that of fatty acid composition, with PC1 changing mainly over time, whereas PC2 represented differences between the two genotypes (Supplementary Figure S7).

### Absolute concentration of lipid species

The above analyses were expressed in mole%. Our mass spectrometry data were also expressed in absolute concentrations (nmol/μl serum, publicly reposited at https://doi.org/10.7910/DVN/5NB36Y) for 370 lipid species. The concentrations of these lipids were impacted by age, species (pig versus ferret), genotype, and sex (Supplementary Figure S8). All 370 were examined for differences between CF and non-CF genotypes, using mixed effects modeling to account for the impact of sex, age, and diet, treating ferret pairings as random effects. For pig, the analysis was underpowered, such that no differences reached statistical significance when accounting for multiple hypothesis testing (Supplementary Figure S9). By contrast, for ferrets, genotype impacted the concentrations of 145 lipids as evidenced by FDR<0.05 (Supplementary Figure S9; see https://doi.org/10.7910/DVN/5NB36Y for tabular list of all comparisons) with 91 of these meeting the stringent Bonferroni criteria of *P*<0.05/370 (Supplementary Figure S9). As evident in the volcano plot (Supplementary Figure S9), the majority of the significantly impacted lipids exhibited a lower concentration in CF compared with non-CF animals. Of the 91 lipids impacted at the Bonferroni significance level, only 3 were increased in CF animals. Interestingly, each of these 3 were saturated non-esterified fatty acids (14:0, 20:0, and 22:0). Similar to the above analyses using mole%, the absolute concentration of the lipid species in ferrets evolved over time, with the differences between genotype persisting over the first weeks of life indicated by parallel changes despite ongoing changes with aging (Supplementary Figure 10S).

### Gene expression

PUFA abnormalities in cultured CF cells have been associated with increased expression of desaturases SCD, FADS1 and FADS2, and elongase ELOVL6 [63,64]. We thus examined the expression of these genes in the liver, considered the dominant organ for PUFA metabolism [65]. Of these, the only one whose expression changed was ELOVL6, which was diminished in CF versus non-CF newborns (Supplementary Figure S11).

## Discussion

CF induces a characteristic disturbance of fatty acid composition in humans, first noted 60 years ago [3]. Despite improvements in nutritional and pulmonary CF care extending life expectancy of humans with CF by decades, these lipid imbalances have persisted [4,5] Prominent derangements commonly observed among polyunsaturated fatty acids in CF include low linoleic acid (the most abundant omega 6 fatty acid) and docosahexaenoic acid (an omega 3 fatty acid) and elevated Mead acid (an omega 9 fatty acid whose synthesis increases during essential fatty acid deficiency). Unlike omega 9 fatty acids, omega 3 and omega 6 fatty acids cannot be synthesized by the body but rather must be acquired by intestinal absorption from dietary sources. Given that CF commonly causes intestinal malabsorption, it has been natural to speculate that CF intestinal disease is an underlying cause of CF fatty acid imbalance. In fact, despite modern pancreatic enzyme replacement therapy, many CF patients exhibit a degree of fat malabsorption [66]. Most CF mice exhibit similar fatty acid abnormalities as humans [11,20,32,33,36,38], despite mice having CF pathology mainly limited to the gastrointestinal tract. Here, we find that two non-rodent models that broadly recapitulate the histopathology of CF also develop fatty acid composition abnormalities that overlap those observed in humans. Furthermore, these animal models enabled us to study the development of fatty acid abnormalities very early in life. Indeed, our data showed that fatty acid composition abnormalities are already present in the liver and serum/plasma on the day of birth, even before feeding. The most prominent of the polyunsaturated fatty acid abnormalities observed in the newborn samples was a relative deficiency of linoleic acid. This was present in both species on the day of birth, was present in a variety of liver phospholipid fractions, and in the serum/plasma. Concordantly, in humans with CF, low linoleic acid is a consistently observed fatty acid abnormality [5] .

Although there were extensive fatty acid composition abnormalities in liver and serum/plasma of newborn animals, these abnormalities were largely absent in the full lipid RBC fraction of newborn ferrets and pigs. This probably reflects that newborn RBC fatty acid composition is highly correlated with maternal fatty acid status/composition [51] the CF and non-CF animals shared the same *in utero* environment. Alternatively, it could be that fatty acid composition imbalance does not exist in CF until birth. The early life fatty acid profile of humans with CF exhibits abnormalities in cord blood plasma phospholipids from newborns (Lloyd-Still et al. 1991) with the profile becoming progressively more abnormal over the first weeks-months of life [67,68]. Birth is coupled to the loss of maternal fuel supply and rapid changes in lipid metabolism [69] including a surge of lipolysis and circulating fatty acids [70]. Likewise, β-oxidation of fatty acids increases after birth due to rising oxygen tension and increased need for endogenous energy production. It is unclear whether the length of time would be sufficient to produce the substantive abnormalities in fatty acid composition that we observed in the liver and serum/plasma of newborn animals. For example, several weeks are required for dietary changes to be reflected in liver phospholipid fatty acid composition in young rats [71]. Based on this one could speculate that our data indicate that fatty acid abnormalities exist before birth in CF.

After the newborn period, we found that the differences between CF and non-CF animal fatty acid composition persisted during early life, despite parallel changes in composition with age, as shown by principal component analysis. This analysis showed a degree of convergence in fatty acid composition between the genotypes approximately 13–20 days of age (Figure 6). We cannot rule out that this was statistical noise, but nonetheless convergence does not persist as RBC fatty acid composition was markedly abnormal in 56–112 days old CF ferrets.

Our data show that a number of fatty acids are impacted in pigs and/or ferrets by CF. The dominant abnormalities most consistently observed in humans, namely diminished linoleic and docosahexaenoic acids and increased Mead acid, were recapitulated. As noted above, most prominent among these were decreases in linoleic acid, in agreement with studies in patients. We also observed decreased docosahexaenoic acid, though this was more prominent in CF ferrets than in CF pigs. Mead acid and palmitoleic acid were increased, just as observed in humans with CF (Rogiers et al. 1980). We observed composition abnormalities in other PUFAs as well. Eicosapentaenoic acid was decreased in CF ferrets, similar to humans with CF [72]. There was considerable variability in the levels of arachidonic acid, similar to humans with CF, where levels are sometimes increased [38] or decreased [10,73]. The dysregulation of cellular arachidonic acid flux in CF, diminished at baseline but with impaired suppression [16], might underlie the varying levels of this fatty acid. Interestingly, dihomo-γ-linolenic acid was decreased in young CF pigs and ferrets, contrary to observed elevations in humans with CF [10,73,74]. However, in our dataset dihomo-γ-linolenic acid was elevated in older CF ferrets. Thus, dihomo-γ-linolenic acid composition may change with age. Our data showed elevation of several saturated and monounsaturated fatty acids, similar to humans with CF [7,72,73,75]. Elevation of oleic acid has been suggested to be compensatory for low linoleic acid, similar to Mead acid compensation for low arachidonic acid [31]. Similarly, elevated palmitoleic acid is a common feature of linoleic acid deficiency [31,68,76]. Overall, our data show that these novel animal models of CF develop the characteristic fatty acid imbalance exhibited by humans with CF, starting early in life.

It is not fully understood which tissues contribute to the fatty acid composition abnormalities in CF. The fatty acid composition abnormalities in CF are described to occur in humans regardless of pancreatic disease [10,31] and also occur in mice which have minimal pancreatic disease [36], indicating that overt pancreatic disease is not required for the lipid imbalance. In humans the lipid abnormalities are more pronounced in patients with mutations associated with a more severe phenotype [31,74,75]. Although chronic pulmonary infection does not develop until some time after birth, pulmonary inflammation is present even in uninfected fetal CF human airway [77]. Interestingly, plasma fatty acid levels, but not relative composition, largely normalized after lung transplant in CF patients in one study [78], whereas a subsequent study found fatty acid composition to be similar to that in non-transplanted CF patients but without shown comparison with healthy references [79]. These results have been interpreted to implicate that severe clinical CF inflammation and/or CF lung disease itself may contribute to the lipid imbalance [24], and studies have also implicated that linoleic acid deficiency is associated with lung dysfunction [5,80,81]. Additionally, cultured pancreatic ductal and bronchial epithelial cells exhibit fatty acid composition abnormalities, suggesting that the lipid imbalance of CF may be cell autonomous [12,63,64,82].

Several molecular mechanisms have been postulated to contribute to the defects in fatty acid composition in CF. The release of arachidonic acid from phospholipids is increased from CF cells [16–19,83], a mechanism that accounts for the increased metabolism of n-6 fatty acids and thus depleted levels of linoleic acid [84]. Since release of arachidonic acid is the rate-limiting step for inflammatory prostanoid formation that would explain the increase of these products [82,85–88]. Our dataset from CF ferrets and pigs is consistent with this mechanism, in that linoleic acid concentrations are profoundly diminished while those of arachidonic acid are highly variable. A separate proposed mechanism involves increased flux of fatty acids through enhanced desaturation and elongation, thus diminishing linoleic acid levels [89]. However, we found no increase in the hepatic expression of several genes involved in PUFA desaturation and elongation when measured in newborns. At this age, we found circulating serum phospholipids to already have altered PUFA ratios. Taken together, this suggests that altered SCD, FADS1, FADS2, and ELOVL6 are not responsible for the early life changes in PUFA status in CF. Rather, abnormalities in expression of these enzymes may be secondary to the PUFA abnormalities, consistent with cell culture studies finding that PUFA supplementation can reverse the excess expression [90]. Interestingly, we observed an increase of the trans isomers of oleic acid and linoleic acid (i.e. increased 18:1t9 and 18:2t6) in the RBCs of CF ferrets. One possible explanation for the increase of trans-fatty acids in CF could be oxidative damage [91], which is generally increased by CF [92] and can catalyze *in vivo* cis/trans isomerization of fatty acids [93]. The need for vitamin E supplementation in CF has long been recognized [8] and is recommended for routine supplementation. Another mechanism that can negatively influence essential fatty acid status in CF is the enteral loss of biliary choline phosphoglycerides due to diminished phospholipase A2 levels and activity [94]. Potentially consistent with this, among the 53 phosphatidylcholine species we quantified in ferret serum by mass spectrometry, all 40 that showed statistical evidence for significant differences between genotypes exhibited diminished concentration in CF serum (details can be found in publicly reposited file serumConcentrationsAllComparisons.tab at https://doi.org/10.7910/DVN/5NB36Y). Note that none of these mechanisms are mutually exclusive, and any combination might contribute to the fatty acid abnormalities in CF. Oral supplementation with docosahexaenoic acid or linoleic acid raises plasma concentrations in CF patients [95,96]. Linoleic acid supplementation restores normal growth to children with CF whereas provision of excess calories alone is not sufficient [96–98]. In contrast, docosahexaenoic acid supplementation in patients with CF has been less impactful [99–101].

CF dramatically altered phospholipid composition in serum of ferrets. Our analysis found no statistically significant changes in phospholipid composition in the serum of CF pigs after accounting for multiple hypothesis testing. However, the pig studies must be interpreted bearing in mind the poor statistical power given that 292 different phospholipid species were examined with only *N* = 5–6 samples per group. Consistent with the overall changes in fatty acid composition, in phospholipids we found decreases in the relative abundance of some with higher unsaturation degrees and overabundance of some with lower degrees of unsaturation.

Our study has both limitations and strengths. Strengths include study of two models that more fully recapitulate human CF pathology than rodent models, the amalgamation of independent results from three laboratories, and the breadth of tissues, lipid classes, and conditions sampled. This enabled divergent statistical approaches: analyses isolating multiple experimental variables (i.e. Figures 2, 4, 5, and Table 2) and analyses jointly examining the data across experimental variables (i.e. Figures 1, 3, 6, and 7). Importantly, our models allowed examination of fatty acid composition prior to first feeding, using a sibling approach such that CF and non-CF had just been born from the same in utero environment. Limitations of our study include that most tissues were not sampled and that most of our analyses only examined relative fatty composition rather than absolute concentrations. For these reasons, we can not state whether there existed whole-body perturbations. Another limitation is pertinent to only those animals that were fed, in that we did not measure the fatty acid composition of their diets or of their stools, and thus cannot determine whether the imbalances might relate to enteral intake or output. Pertinent to newborns, we did not measure fatty acid composition in the mothers blood or in their milk. However, we used a sibling control strategy such that the same *in utero* and *post utero* environments were experienced between genotypes. Another limitation of our study is that we did not measure human CF samples to allow direct comparison in our hands, although one of our labs has a long history measuring fatty acid composition in human CF samples (Strandvik et al. 2001 [75]) .

In summary, we report that two new animal CF models recapitulate key fatty acid imbalances found in humans with CF, particularly relative reductions in linoleic acid and increases in Mead acid. Likewise, the varying results for arachidonic and docosahexaenoic acids in our models are similar to variances found in human CF studies. These fatty acid composition abnormalities exist in liver and plasma phospholipids before first feeding and may even exist *in utero*, and then rapidly become more widespread. These results indicate that the lipid abnormalities are fundamental to loss of functional CFTR.

## Clinical perspectives

- The origins and physiological mechanisms underlying the fatty acid imbalance that exists in CF are poorly understood.
- Both pigs and ferrets with cystic fibrosis recapitulate many aspects of the fatty acid imbalance found in humans with cystic fibrosis. The imbalance is present at birth, even before feeding.
- Interspecies conservation suggests that the fatty imbalance of cystic fibrosis is inherent to the disease. That the abnormalities precede feeding suggests that dietary measures alone will not address the mechanisms of the imbalance.

## Supplementary Material

Supplementary Figures S1-S11 and Tables S1-S4

## Data Availability

All data presented herein are available publicly at https://doi.org/10.7910/DVN/5NB36Y

## Abbreviations

BHT: butylated hydroxytoluene
CF: cystic fibrosis
CFTR: cystic fibrosis transmembrane conductance regulator
FDR: false discovery rate
FAME: fatty acid methyl esters
PUFA: polyunsaturated fatty acid
RBC: red blood cell
