# Supplementary material for: The fatty acid imbalance of cystic fibrosis exists at birth independent of feeding in pig and ferret models
Source: Clin Sci (Lond). 2022 Dec 13;136(24):1773–91. doi: 10.1042/CS20220450 (PMC9747517; doi:10.1042/CS20220450)

Table 1S: Primer sequences. The table lists the target species, gene, forward and reverse primer sequences (5' to 3'), amplicon size, and relevant NCBI accession.

Genes of Interest

Species	Gene	Forward	Reverse	Size	Accession
Ferret	SCD	GTGATGTTCCAGAGAAGGTACTACAA	AACAGAGTGGCGACGAACAA	125	XM_004749483.3
Ferret	FADS1	AAGCATGGCATAGAGTACCAGTC	CCAGACTCCTTCAGTGAGTAGAC	77	XM_004770330.3
Ferret	ELOVL6	CGAAGTTTGAACTGAGGAAGCC	CGAAGAGCACCGAATATACTGAAG	76	XM_045085544.1
Ferret	FADS2	ATGGCAAGAACTCCCAGATCAC	AGAAGAGGATGAACCAGGCAATG	150	XM_013044579.2
Pig	SCD	ACACTTGGGAGCCCTGTATG	GGGCAGTCGAGCTTTGTAAG	154	NM_213781.1
Pig	FADS2	AGGACATGAACCTGTTCGAGAGCA	TTCTTGTAAACAGAGAGATGGCCG	208	NM_001171750.1

References Genes

Species	Gene	Forward	Reverse	Size	Accession
Ferret	RPS18	CTCACTGAGGATGAGGTGGAAC	ACCAGTCTGGGATCTTATATTGGC	73	XM_045074698.1
Ferret	RPLP0	CTTCTTCCAGGCTTTAGGCATCA	CTGCACATCACTCAGGATTTCAA	70	XM_045073641.1
Pig	ACTB	CTGCGGCATCCACGAACCT	GTGATCTCCTTCTGCATCCTGTC	141	XM_021086047.1

Table 2S: Fatty acids measured per lab and their unification for composition analysis. For unsaturated fatty acids measured by mass spectrometry (“Kansas”), these were unified with their corresponding fatty acids measured by gas chromatography when there were no identified alternate positional isoforms.

Unified	Sweden	Kansas	Iowa
14:0	14:0	14:0	
16:0	16:0	16:0	16:0
16:1n7	16:1n7	16:1	16:1n7
18:0	18:0	18:0	18:0
18:1n7			18:1n7
18:1n9	18:1n9		18:1c9
18:1t9			18:1t9
18:2n6	18:2n6	18:2	18:2c6
18:2t6			18:2t6
18:3n3	18:3n3		18:3n3
18:3n6	18:3n6		18:3n6
18:4n3			18:4n3
20:0	20:0	20:0	20:0
20:1n9		20:1	20:1n9
20:2n6	20:2n6	20:2	
20:3n6	20:3n6		20:3n6
20:3n9	20:3n9		
20:4n6	20:4n6	20:4	20:4n6
20:5n3	20:5n3	20:5	20:5n3
22:0	22:0	22:0	22:0
22:1n9		22:1	22:1n9
22:4n6	22:4n6	22:4	22:4n6
22:5n3	22:5n3		22:5n3
22:5n6	22:5n6		
22:6n3	22:6n3	22:6	22:6n3
24:0	24:0		24:0
24:1n9	24:1n9		24:1n9

Table 3S: Fatty acids measured per lab omitted from unified analysis. The mean ± s.e.m. mole% for each fatty acid measurement across the dataset is shown in parentheses. Footnotes indicate the reason(s) each fatty acid was omitted from the unified dataset.

Not unified	Sweden	Kansas	Iowa
12:0	12:0[a] (0 ± 0)		
14:1n5	14:1n5[a] (0.0035 ± 0.0007)		
18:1		18:1[b,c] (25.64± 1.05)	
18:3		18:3[b] (1.37 ± 0.19)	
20:2n6+20:3n9			20:2n6+20:3n9[d] (1.86 ± 0.12)
20:3n3			20:3n3[a] (0.057 ± 0.013)
20:3		20:3[b] (4.02 ± 0.37)	
21:0		21:0[a] (0.024 ± 0.024)	
21:1		21:1[b] (2.64 ± 0.23)	
22:2n6			22:2n6[a] (0.054 ± 0.004)
22:3n3			22:3n3[a] (0.061 ± 0.002)
22:5		22:5[b] (1.64 ± 0.22)	

[a] Fatty acid of very minimal abundance (<0.1%) measured only in one laboratory. [b] Likely mix of two or more unsaturation positions of non-trivial relative abundance. [c] Porcine red blood cells and serum can contain non-trivial (2-7 mole%) amounts of 18:1n-7 (see PMID: 22223579 & 19434740). [d] These two fatty acids could not be resolved from each other.

Table 4S: Fatty acids whose composition was significantly impacted by cystic fibrosis. Of 559 pairwise comparisons between CF and non-CF, 70 met strict criteria for statistical significance of FDR<0.05 accounting for multiple hypothesis testing. Mean ± SEM are shown for non-CF and CF. Lipid class abbreviations: L total lipid; CE cholesterol esters, NEFA non-esterified fatty acids; PL general phospholipids; PC phosphatidylcholine; PE phosphatidylethanolamine; PI phosphatidylinositol; PS phosphatidylserine; LPC lysophosphatidylcholines; LPE lysophosphatidylethanolamine; NEFA non-esterified fatty acids; SM2 sphingomyelin 2. Trans fatty acids are indicated by a "t". Newborns 0-1 were days old; preweans 2-42 days old; juvenile, 43-150 days old. The table is presented twice, first sorted by fatty acid, and second sorted by species. ([a]) Unsaturated fatty acids without an omega indicator were measured by mass spectrometry and thus positional information was not determined. Generally, 18:1, 18:3, 22:5 may be a mixture of different omega isomers. By contrast, the following species are likely nearly entirely comprised of the indicated omega isomer: 16:1 ≅ 16:1n-7; 18:2 ≅ 18:2n-6; 20:1 ≅ 20:1n-9; 20:4 ≅ 20:4n-6; 20:5 ≅ 20:5n-3; 22:6 ≅ 22:6n-3.

Sorted by fatty acid

Fatty Acid	Tissue	Class	CF	non-CF	Age	Species	N	FDR
14:0	Plasma	NEFA	3.14±0.26	1.84±0.15	Newborn	Ferret	5,5	0.02619
14:0	Plasma	NEFA	2.89±0.17	1.59±0.25	Prewean	Ferret	6,6	0.01851
16:0	Liver	L	18.49±0.26	20.72±0.37	Newborn	Pig	13,7	0.00319
16:0	Liver	SM2	28.95±1.69	37.9±1.46	Newborn	Pig	13,7	0.02491
16:0	Plasma	LPC	57.36±2.44	46.03±1.1	Prewean	Ferret	6,6	0.01926
16:0	RBC	L	27.86±0.49	24.55±0.27	Juvenile	Ferret	5,6	0.00413
16:1 [a]	Plasma	CE	16.95±0.33	12.44±0.8	Newborn	Pig	6,5	0.00596
16:1 [a]	Plasma	LPC	5.45±0.23	3.37±0.44	Newborn	Pig	6,5	0.01851
16:1n7	Liver	L	4.9±0.2	3.72±0.29	Newborn	Pig	13,7	0.03008
16:1n7	Liver	PC	5.74±0.21	3.96±0.29	Newborn	Pig	13,7	0.00319
18:0	RBC	L	13.61±0.36	15.73±0.38	Juvenile	Ferret	5,6	0.03008
18:1 [a]	Plasma	LPE	49.46±1.79	20.41±3.54	Newborn	Ferret	5,5	0.00319
18:1n7	Liver	L	14.24±0.51	11.62±0.43	Newborn	Ferret	17,24	0.00596
18:1n7	RBC	L	4.37±0.27	2.86±0.07	Juvenile	Ferret	5,6	0.00509
18:2 [a]	Plasma	NEFA	4.18±0.31	9.44±0.76	Newborn	Ferret	5,5	0.00429
18:2 [a]	RBC	L	5.09±0.52	11.36±0.95	Juvenile	Ferret	5,6	0.00646
18:2n6	Liver	L	4.91±0.15	8.29±0.9	Newborn	Pig	13,7	0.00319
18:2n6	Liver	L	4.54±0.11	5.31±0.14	Newborn	Pig	4,5	0.03386
18:2n6	Liver	PC	4.55±0.16	9.39±1.2	Newborn	Pig	13,7	0.00244
18:2n6	Liver	PE	1.9±0.07	4.71±0.62	Newborn	Pig	13,7	0.00071
18:2n6	Liver	PI	0.34±0.03	1.01±0.27	Newborn	Pig	12,7	0.03967
18:2n6	Liver	PS	0.85±0.05	1.92±0.33	Newborn	Pig	13,7	0.00607
18:2n6	Liver	SM2	0.3±0.02	0.55±0.06	Newborn	Pig	13,7	0.00570
18:2n6	Serum	PL	4.03±0.4	15.89±1.58	Newborn	Pig	13,7	0.00001
18:2t6	RBC	L	0.18±0.02	0.05±0.01	Juvenile	Ferret	5,6	0.00319
18:3 [a]	Plasma	NEFA	1.27±0.07	1.61±0.03	Newborn	Ferret	5,5	0.03204
18:3n6	RBC	L	0.17±0.01	0.1±0.01	Juvenile	Ferret	5,6	0.01442
18:4n3	RBC	L	0.56±0.04	0.4±0.01	Juvenile	Ferret	5,6	0.03256
20:0	Plasma	NEFA	2.01±0.18	0.77±0.08	Newborn	Ferret	5,5	0.00509
20:0	Serum	PL	1.81±0.16	0.8±0.15	Newborn	Pig	13,7	0.00882

20:1 [a]	Plasma	NEFA	1.4±0.08	0.61±0.07	Newborn	Ferret	5,5	0.00319
20:1n9	RBC	L	0.65±0.02	0.56±0.01	Newborn	Ferret	9,13	0.02143
20:2n6	Liver	L	0.06±0	0.18±0.03	Newborn	Pig	13,7	0.00347
20:2n6	Liver	PC	0.07±0	0.18±0.03	Newborn	Pig	13,7	0.00299
20:2n6	Liver	PE	0.05±0	0.2±0.03	Newborn	Pig	13,7	0.00107
20:2n6	Serum	PL	0.08±0.01	0.21±0.03	Newborn	Pig	13,7	0.00063
20:2n6	RBC	L	1.6±0.24	0.41±0.02	Juvenile	Ferret	5,6	0.00596
20:3n6	Liver	PS	0.77±0.05	1.11±0.09	Newborn	Pig	13,7	0.01851
20:3n6	RBC	L	0.87±0.04	0.4±0.02	Juvenile	Ferret	5,6	0.00026
20:3n9	Liver	L	1.69±0.16	0.94±0.12	Newborn	Pig	13,7	0.03936
20:3n9	Liver	PC	1.24±0.12	0.61±0.09	Newborn	Pig	13,7	0.02083
20:3n9	Liver	PI	8.24±0.66	4.65±0.65	Newborn	Pig	12,7	0.02348
20:4 [a]	Plasma	LPC	4.06±0.72	8.98±0.58	Newborn	Ferret	5,5	0.00954
20:4 [a]	Plasma	LPC	3.69±0.54	9.15±0.68	Prewean	Ferret	6,6	0.00319
20:4n6	Liver	PE	29.31±0.38	24.44±0.66	Newborn	Pig	13,7	0.00026
20:5 [a]	Plasma	CE	2.19±0.22	4.86±0.36	Newborn	Ferret	5,5	0.00452
20:5 [a]	Plasma	CE	2.86±0.28	4.99±0.2	Prewean	Ferret	6,6	0.00319
20:5 [a]	Plasma	LPC	0.35±0.1	0.92±0.05	Newborn	Ferret	5,5	0.01516
20:5n3	Liver	L	0.16±0.01	0.09±0.01	Newborn	Pig	13,7	0.01752
20:5n3	Liver	PC	0.17±0.01	0.08±0.02	Newborn	Pig	13,7	0.00429
20:5n3	Liver	PE	0.23±0.01	0.15±0.02	Newborn	Pig	13,7	0.04568
20:5n3	RBC	L	0.28±0.1	1.02±0.11	Juvenile	Ferret	5,6	0.01443
22:1n9	RBC	L	0.22±0.02	0.15±0.01	Juvenile	Ferret	5,6	0.03008
22:4n6	Liver	PC	0.47±0.05	0.27±0.02	Newborn	Pig	4,5	0.03311
22:4n6	Liver	PE	0.74±0.05	0.46±0.04	Newborn	Pig	4,5	0.02619
22:4n6	Liver	PS	0.37±0.04	0.61±0.08	Newborn	Pig	13,7	0.04914
22:4n6	RBC	L	3.3±0.32	1.68±0.1	Juvenile	Ferret	5,6	0.00796
22:5 [a]	Plasma	LPC	0.43±0.13	1.09±0.05	Newborn	Ferret	5,5	0.01752
22:5 [a]	Plasma	LPC	0.27±0.06	0.81±0.08	Prewean	Ferret	6,6	0.00635
22:5n3	Liver	LPC	0.77±0.06	1.78±0.26	Newborn	Pig	13,7	0.00374
22:5n3	Serum	PL	0.35±0.03	0.49±0.02	Newborn	Pig	13,7	0.04834
22:5n6	Liver	PS	4.27±0.36	7.42±0.8	Newborn	Pig	13,7	0.00850
22:6 [a]	Plasma	LPC	0.88±0.24	2.7±0.33	Newborn	Ferret	5,5	0.02083
22:6 [a]	Plasma	LPC	0.7±0.16	2.57±0.28	Prewean	Ferret	6,6	0.00413
22:6 [a]	Plasma	LPE	8.87±2.75	30.17±3.88	Newborn	Ferret	5,5	0.02143
22:6 [a]	Plasma	LPE	8.55±1.86	32.46±3.67	Prewean	Ferret	6,6	0.00413
22:6n3	Liver	LPC	2.18±0.22	4.74±0.63	Newborn	Pig	13,7	0.00413
24:0	Serum	PL	0.7±0.04	0.42±0.07	Newborn	Pig	13,7	0.01693
24:1n9	Serum	PL	3.13±0.21	1.47±0.27	Newborn	Pig	13,7	0.00413
24:1n9	RBC	L	2.11±0.07	1.21±0.05	Juvenile	Ferret	5,6	0.00026

Sorted by species

**Fatty Acid**	**Tissue**	**Class**	**CF**	**non-CF**	**Age**	**Species**	**N**	**FDR**
14:0	Plasma	NEFA	3.14±0.26	1.84±0.15	Newborn	Ferret	5,5	0.02619
14:0	Plasma	NEFA	2.89±0.17	1.59±0.25	Prewean	Ferret	6,6	0.01851
16:0	Plasma	LPC	57.36±2.44	46.03±1.1	Prewean	Ferret	6,6	0.01926

16:0	RBC	L	27.86±0.49	24.55±0.27	Juvenile	Ferret	5,6	0.00413
18:0	RBC	L	13.61±0.36	15.73±0.38	Juvenile	Ferret	5,6	0.03008
18:1	Plasma	LPE	49.46±1.79	20.41±3.54	Newborn	Ferret	5,5	0.00319
18:1n7	Liver	L	14.24±0.51	11.62±0.43	Newborn	Ferret	17,24	0.00596
18:1n7	RBC	L	4.37±0.27	2.86±0.07	Juvenile	Ferret	5,6	0.00509
18:2	Plasma	NEFA	4.18±0.31	9.44±0.76	Newborn	Ferret	5,5	0.00429
18:2	RBC	L	5.09±0.52	11.36±0.95	Juvenile	Ferret	5,6	0.00646
18:2t6	RBC	L	0.18±0.02	0.05±0.01	Juvenile	Ferret	5,6	0.00319
18:3	Plasma	NEFA	1.27±0.07	1.61±0.03	Newborn	Ferret	5,5	0.03204
18:3n6	RBC	L	0.17±0.01	0.1±0.01	Juvenile	Ferret	5,6	0.01442
18:4n3	RBC	L	0.56±0.04	0.4±0.01	Juvenile	Ferret	5,6	0.03256
20:0	Plasma	NEFA	2.01±0.18	0.77±0.08	Newborn	Ferret	5,5	0.00509
20:1	Plasma	NEFA	1.4±0.08	0.61±0.07	Newborn	Ferret	5,5	0.00319
20:1n9	RBC	L	0.65±0.02	0.56±0.01	Newborn	Ferret	9,13	0.02143
20:2n6	RBC	L	1.6±0.24	0.41±0.02	Juvenile	Ferret	5,6	0.00596
20:3n6	RBC	L	0.87±0.04	0.4±0.02	Juvenile	Ferret	5,6	0.00026
20:4	Plasma	LPC	4.06±0.72	8.98±0.58	Newborn	Ferret	5,5	0.00954
20:4	Plasma	LPC	3.69±0.54	9.15±0.68	Prewean	Ferret	6,6	0.00319
20:5	Plasma	CE	2.19±0.22	4.86±0.36	Newborn	Ferret	5,5	0.00452
20:5	Plasma	CE	2.86±0.28	4.99±0.2	Prewean	Ferret	6,6	0.00319
20:5	Plasma	LPC	0.35±0.1	0.92±0.05	Newborn	Ferret	5,5	0.01516
20:5n3	RBC	L	0.28±0.1	1.02±0.11	Juvenile	Ferret	5,6	0.01443
22:1n9	RBC	L	0.22±0.02	0.15±0.01	Juvenile	Ferret	5,6	0.03008
22:4n6	RBC	L	3.3±0.32	1.68±0.1	Juvenile	Ferret	5,6	0.00796
22:5	Plasma	LPC	0.43±0.13	1.09±0.05	Newborn	Ferret	5,5	0.01752
22:5	Plasma	LPC	0.27±0.06	0.81±0.08	Prewean	Ferret	6,6	0.00635
22:6	Plasma	LPC	0.88±0.24	2.7±0.33	Newborn	Ferret	5,5	0.02083
22:6	Plasma	LPC	0.7±0.16	2.57±0.28	Prewean	Ferret	6,6	0.00413
22:6	Plasma	LPE	8.87±2.75	30.17±3.88	Newborn	Ferret	5,5	0.02143
22:6	Plasma	LPE	8.55±1.86	32.46±3.67	Prewean	Ferret	6,6	0.00413
24:1n9	RBC	L	2.11±0.07	1.21±0.05	Juvenile	Ferret	5,6	0.00026
16:0	Liver	L	18.49±0.26	20.72±0.37	Newborn	Pig	13,7	0.00319
16:0	Liver	SM2	28.95±1.69	37.9±1.46	Newborn	Pig	13,7	0.02491
16:1	Plasma	CE	16.95±0.33	12.44±0.8	Newborn	Pig	6,5	0.00596
16:1	Plasma	LPC	5.45±0.23	3.37±0.44	Newborn	Pig	6,5	0.01851
16:1n7	Liver	L	4.9±0.2	3.72±0.29	Newborn	Pig	13,7	0.03008
16:1n7	Liver	PC	5.74±0.21	3.96±0.29	Newborn	Pig	13,7	0.00319
18:2n6	Liver	L	4.91±0.15	8.29±0.9	Newborn	Pig	13,7	0.00319
18:2n6	Liver	L	4.54±0.11	5.31±0.14	Newborn	Pig	4,5	0.03386
18:2n6	Liver	PC	4.55±0.16	9.39±1.2	Newborn	Pig	13,7	0.00244
18:2n6	Liver	PE	1.9±0.07	4.71±0.62	Newborn	Pig	13,7	0.00071
18:2n6	Liver	PI	0.34±0.03	1.01±0.27	Newborn	Pig	12,7	0.03967
18:2n6	Liver	PS	0.85±0.05	1.92±0.33	Newborn	Pig	13,7	0.00607
18:2n6	Liver	SM2	0.3±0.02	0.55±0.06	Newborn	Pig	13,7	0.00570
18:2n6	Serum	PL	4.03±0.4	15.89±1.58	Newborn	Pig	13,7	0.00001
20:0	Serum	PL	1.81±0.16	0.8±0.15	Newborn	Pig	13,7	0.00882
20:2n6	Liver	L	0.06±0	0.18±0.03	Newborn	Pig	13,7	0.00347

20:2n6	Liver	PC	0.07±0	0.18±0.03	Newborn	Pig	13,7	0.00299
20:2n6	Liver	PE	0.05±0	0.2±0.03	Newborn	Pig	13,7	0.00107
20:2n6	Serum	PL	0.08±0.01	0.21±0.03	Newborn	Pig	13,7	0.00063
20:3n6	Liver	PS	0.77±0.05	1.11±0.09	Newborn	Pig	13,7	0.01851
20:3n9	Liver	L	1.69±0.16	0.94±0.12	Newborn	Pig	13,7	0.03936
20:3n9	Liver	PC	1.24±0.12	0.61±0.09	Newborn	Pig	13,7	0.02083
20:3n9	Liver	PI	8.24±0.66	4.65±0.65	Newborn	Pig	12,7	0.02348
20:4n6	Liver	PE	29.31±0.38	24.44±0.66	Newborn	Pig	13,7	0.00026
20:5n3	Liver	L	0.16±0.01	0.09±0.01	Newborn	Pig	13,7	0.01752
20:5n3	Liver	PC	0.17±0.01	0.08±0.02	Newborn	Pig	13,7	0.00429
20:5n3	Liver	PE	0.23±0.01	0.15±0.02	Newborn	Pig	13,7	0.04568
22:4n6	Liver	PC	0.47±0.05	0.27±0.02	Newborn	Pig	4,5	0.03311
22:4n6	Liver	PE	0.74±0.05	0.46±0.04	Newborn	Pig	4,5	0.02619
22:4n6	Liver	PS	0.37±0.04	0.61±0.08	Newborn	Pig	13,7	0.04914
22:5n3	Liver	LPC	0.77±0.06	1.78±0.26	Newborn	Pig	13,7	0.00374
22:5n3	Serum	PL	0.35±0.03	0.49±0.02	Newborn	Pig	13,7	0.04834
22:5n6	Liver	PS	4.27±0.36	7.42±0.8	Newborn	Pig	13,7	0.00850
22:6n3	Liver	LPC	2.18±0.22	4.74±0.63	Newborn	Pig	13,7	0.00413
24:0	Serum	PL	0.7±0.04	0.42±0.07	Newborn	Pig	13,7	0.01693
24:1n9	Serum	PL	3.13±0.21	1.47±0.27	Newborn	Pig	13,7	0.00413

Figure 1S: Early fatty acid composition abnormalities induced by CF. Fatty acid composition was measured in mole% in the serum and plasma of never-fed newborn pigs. The impact of CF versus non-CF status on fatty acid composition was determined using linear mixed effect modeling treating subjects and lab of analysis as random effects. Linear modeling was used for those fatty acids for which there were not per-subject and per-lab repeated measures. Significance: black FDR<0.05, orange FDR>0.05 and p<0.05, light blue p>0.05. The number of measurements (n) underlying each fatty acid shown on the right side of the graph.

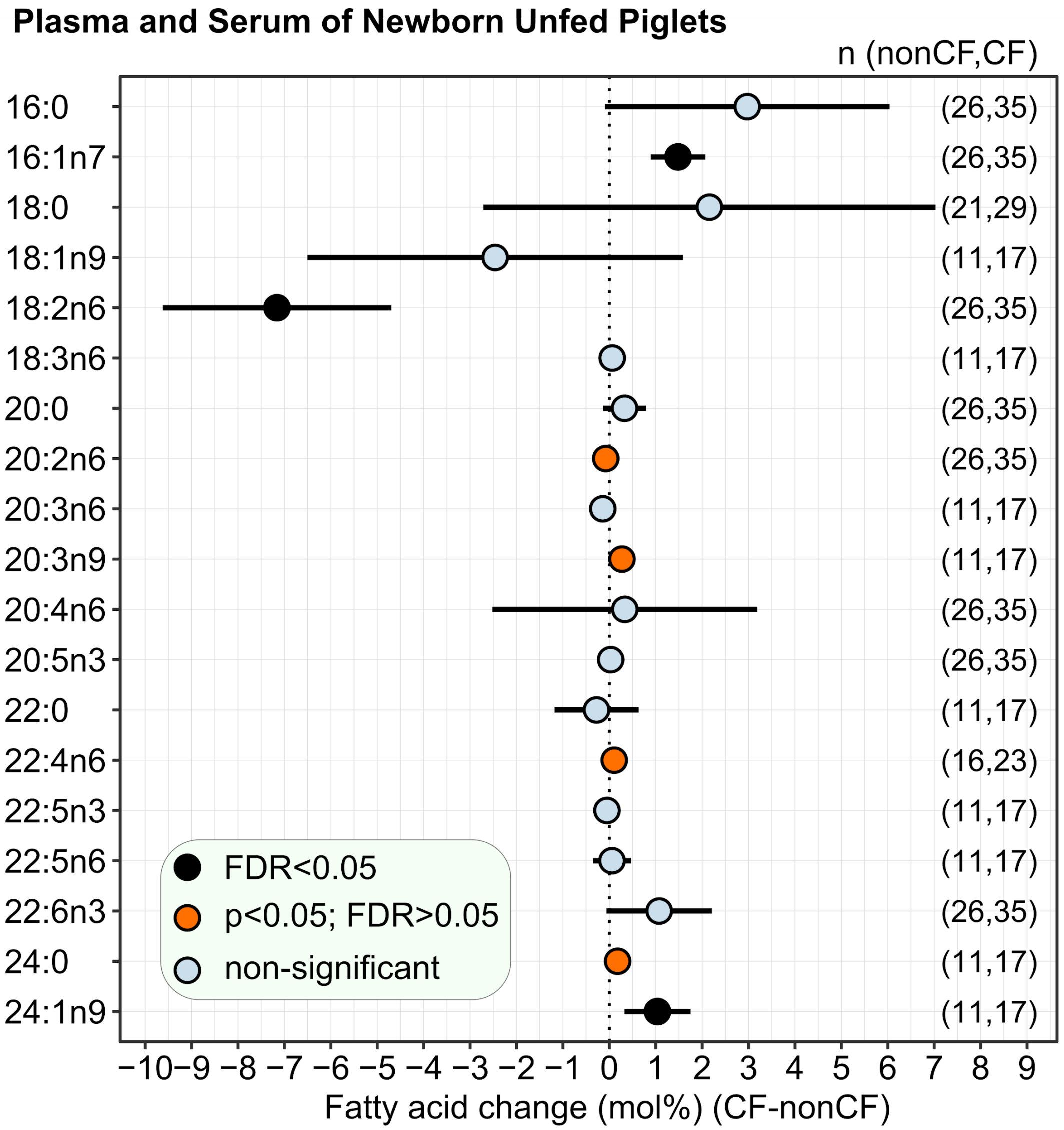

Figure 2S: Ferret plasma lipid fatty acid composition changes over the first 3 weeks of life, examined by lipid class. N=11 ferrets per genotype, without repeated sampling, 1-4 samples per time point. Principal component analysis is shown, plotting the changes in fatty acid profile of CF (red) versus non-CF (blue) ferrets. Four lipid classes were studied: lysophosphatidylcholine LPC, lysophosphatidylethanolamine LPE, cholesterol esters CE, and non-esterified fatty acids NEFA. The age in days is indicated for each point on the graph. The top graphs represent all lipid classes analyzed jointly, and thus are exactly identical to figure 5. The subsequent graphs represent the impact of each isolated lipid class on the principal component coordinates. LPC and LPE are on this page; CE and NEFA on the next page. For each row, the left-sided and right-sided graphs are identical but just shown at different “magnifications”. The left-sided graphs have the identical scale as the all-class analysis to allow comparison, whereas the right-sided graphs are scaled individually “i.e. magnified”.

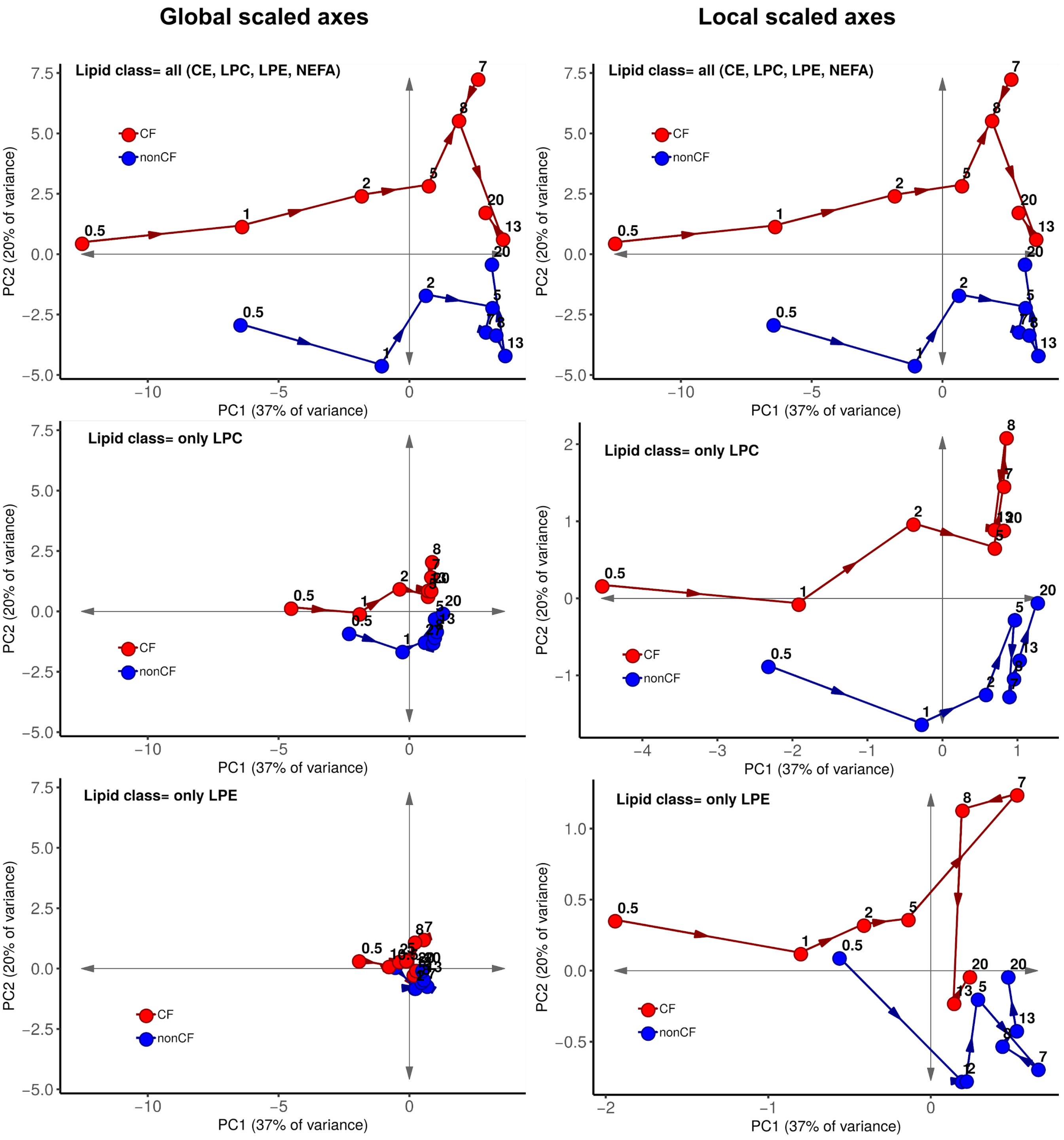

Figure 2S (continued):

Global scaled axes | **Local scaled axes**

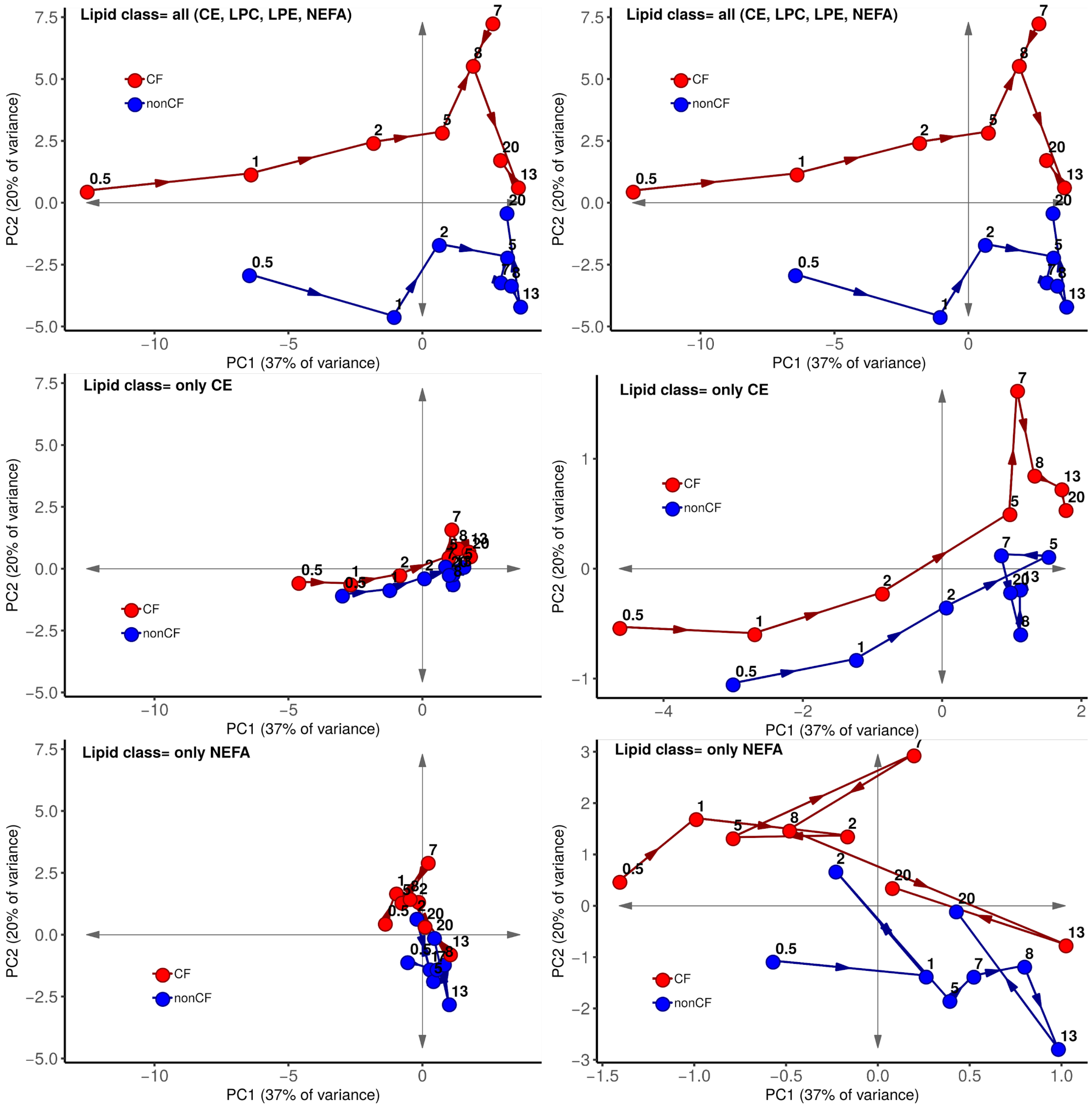

Figure 3S: Ferret plasma lipid fatty acid composition changes over the first 3 weeks of life. N=11 ferrets per genotype, without repeated sampling, 1-4 samples per time point. Changes over time in composition for the top positive and negative contributors to each principal component of Figure 5 are shown.

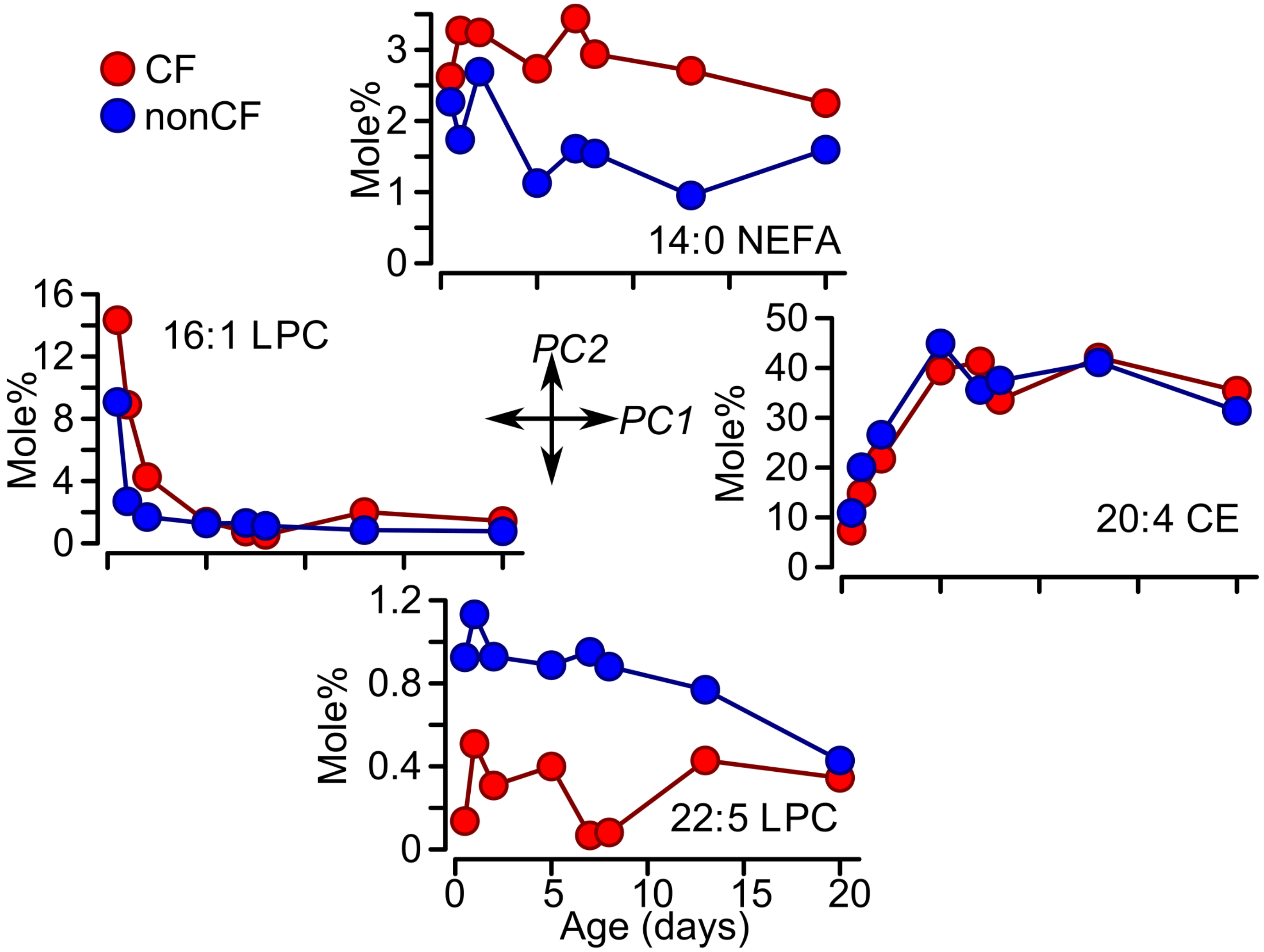

Figure 4S: Relative influence of genotype (CF versus non-CF) and other factors on serum phospholipid composition. The composition of 292 phospholipids was subject to Factor Analysis on Mixed Data. The resulting 2 principal dimensions in log10 space are shown, with the explained variance per axis noted in parentheses.

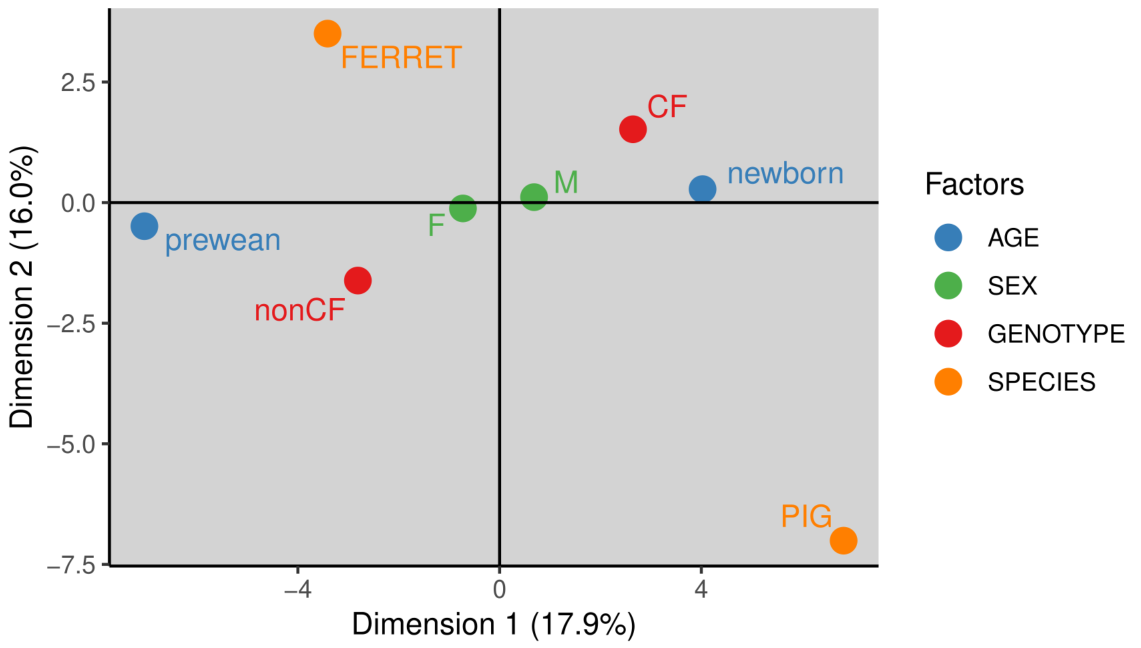

Figure 5S: The impact of CF on composition of 292 phospholipids as assessed by serum samples collected from pigs (A) or ferrets (B). The volcano plots show changes in individual phospholipids between CF and non-CF samples, with significance determined by linear mixed effects modeling. The x-axis indicates the relative genotype effect in mole% divided by the mean mole% across the samples. N= 5 versus 6 (A), and 11 versus 11 (B). Comparisons reaching only nominal significance with p<0.05 are orange; those also reaching a false discovery rate (FDR) of <0.05 are black; those also reaching Bonferroni adjusted significance of p<0.05/292 are red.

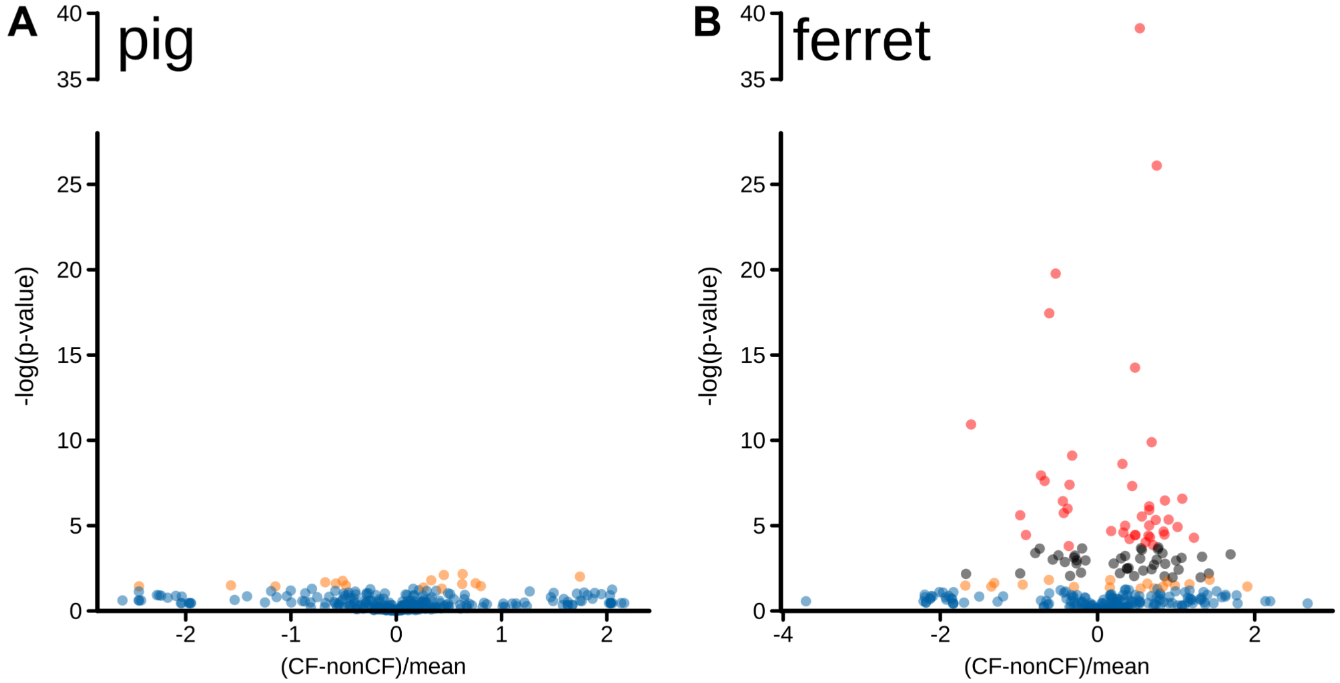

Figure 6S: Phospholipids highly impacted by CF in ferret serum. The 41 phospholipids that were significantly ($p<0.05/292$) impacted by CF are shown. Mean effects and 95% confidence intervals (points and bars) are shown and account for multiple co-variates as described in methods. N=11 non-CF and N=11 CF independent samples.

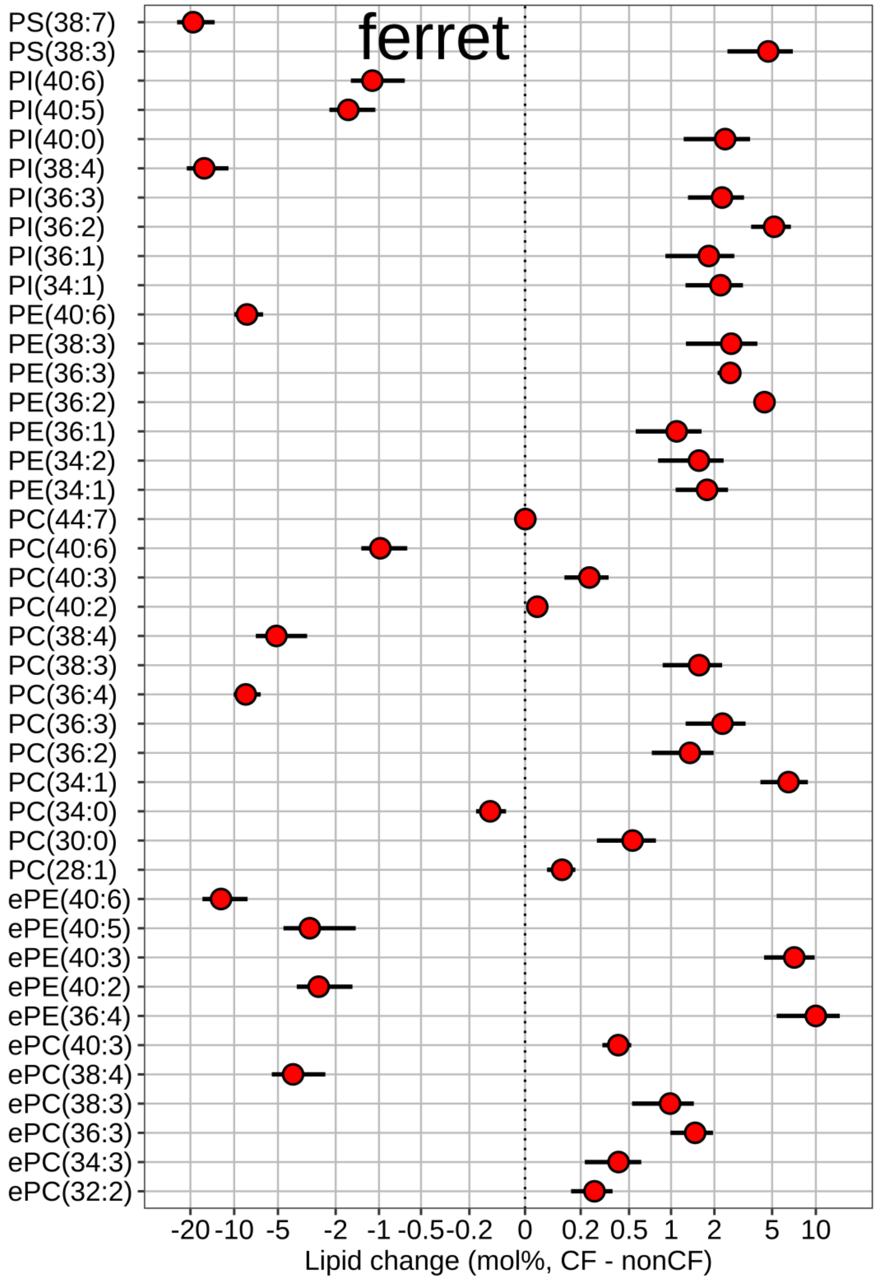

Figure 7S: Ferret plasma phospholipid composition changes over the first 3 weeks of life. N=11 ferrets per genotype, without repeated sampling, 1-4 samples per time point, 292 phospholipid species quantified. Principal component analysis, plotting the changes in fatty acid profile of CF (red) versus non-CF (blue) ferrets over time. The age in days for sample collection is indicated for each point on the graph.

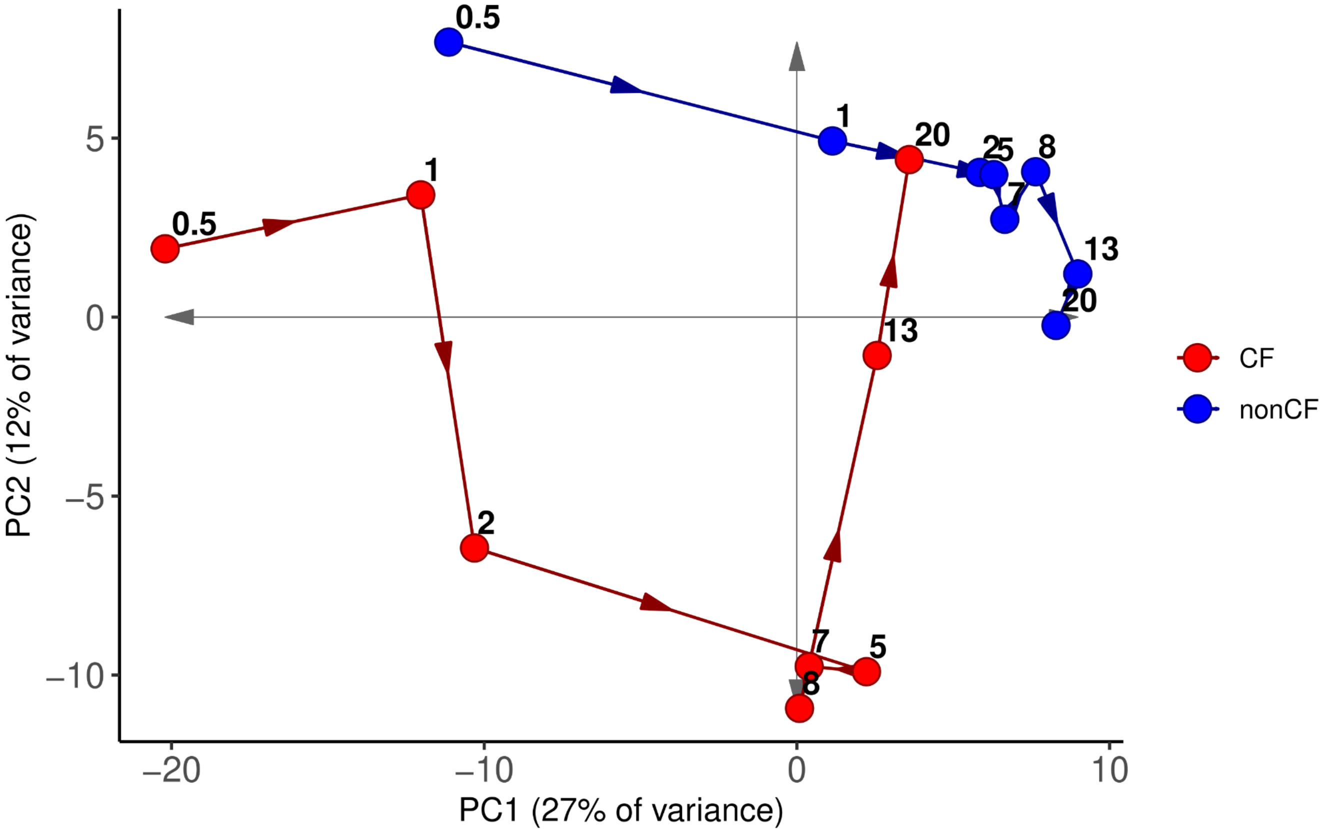

Figure 8S: Relative influence of genotype (CF versus non-CF) and other factors on serum lipid concentrations measured by mass spectrometry. The concentrations of 352-370 serum lipids were measured in serum from 22 ferrets (11 CF, 11 non-CF) and from 11 pigs (6 CF, 5 non-CF) and were subjected to Factor Analysis on Mixed Data. The resulting 2 principal dimensions in log10 space are shown, with the explained variance per axis noted in parentheses.

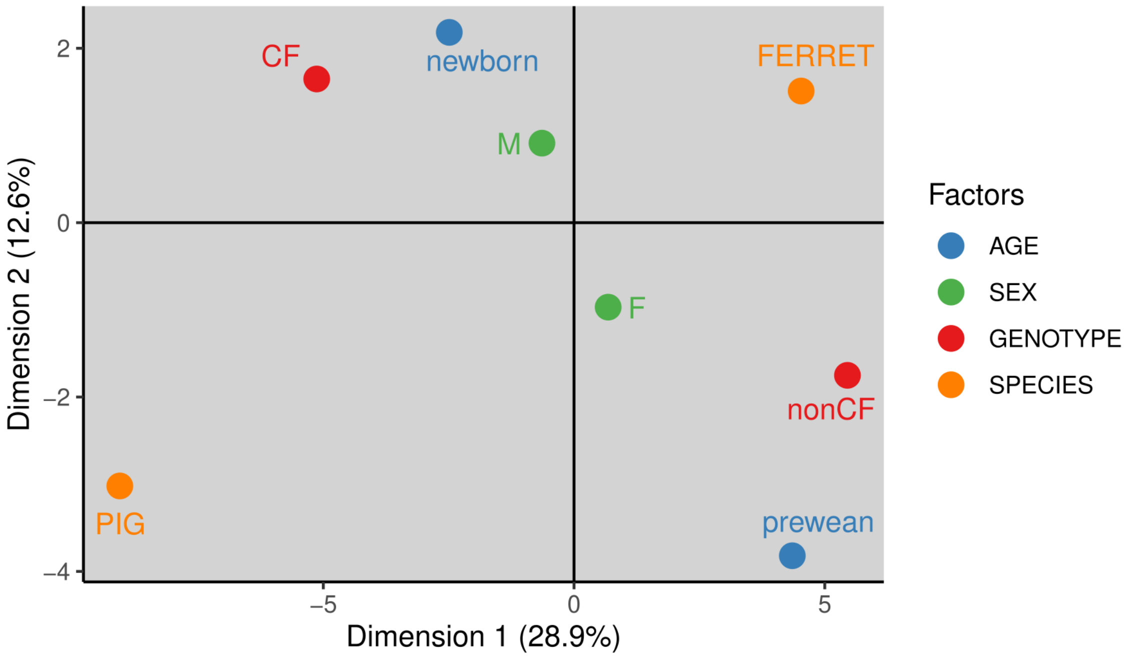

Figure 9S: The impact of CF on concentrations of lipids in serum samples collected from pigs (A) or ferrets (B). The volcano plots show changes in the concentration of individual serum lipid species between CF and non-CF samples, with significance determined by linear mixed effects modeling. The x-axis indicates the relative genotype effect difference in concentration (normalized by the mean concentration across the samples). Examination of 370 lipids with N= 5 versus 6 **(A)**, and examination of 352 lipids with N= 11 versus 11 **(B)**. Comparisons reaching only nominal significance with $p<0.05$ are orange; those also reaching a false discovery rate (FDR) of <0.05 are black; those also reaching Bonferroni adjusted significance of $p<0.05/370$ are red.

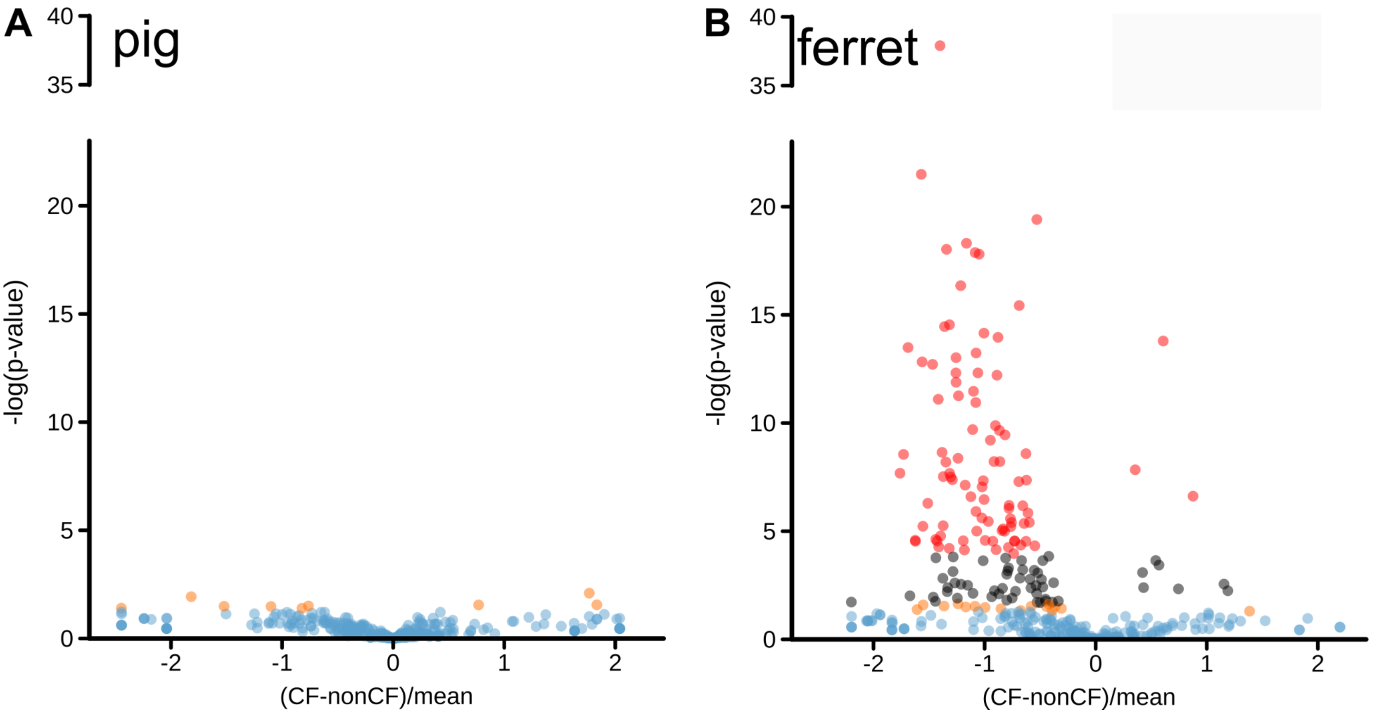

Figure 10S: Changes in lipid species concentrations in ferret plasma over the first 3 weeks of life. N=11 ferrets per genotype, without repeated sampling, 1-4 samples per time point, 370 lipid species quantified. Principal component analysis, plotting the changes in lipid concentration profile of CF (red) versus non-CF (blue) ferrets over time. The age in days for sample collection is indicated for each point on the graph.

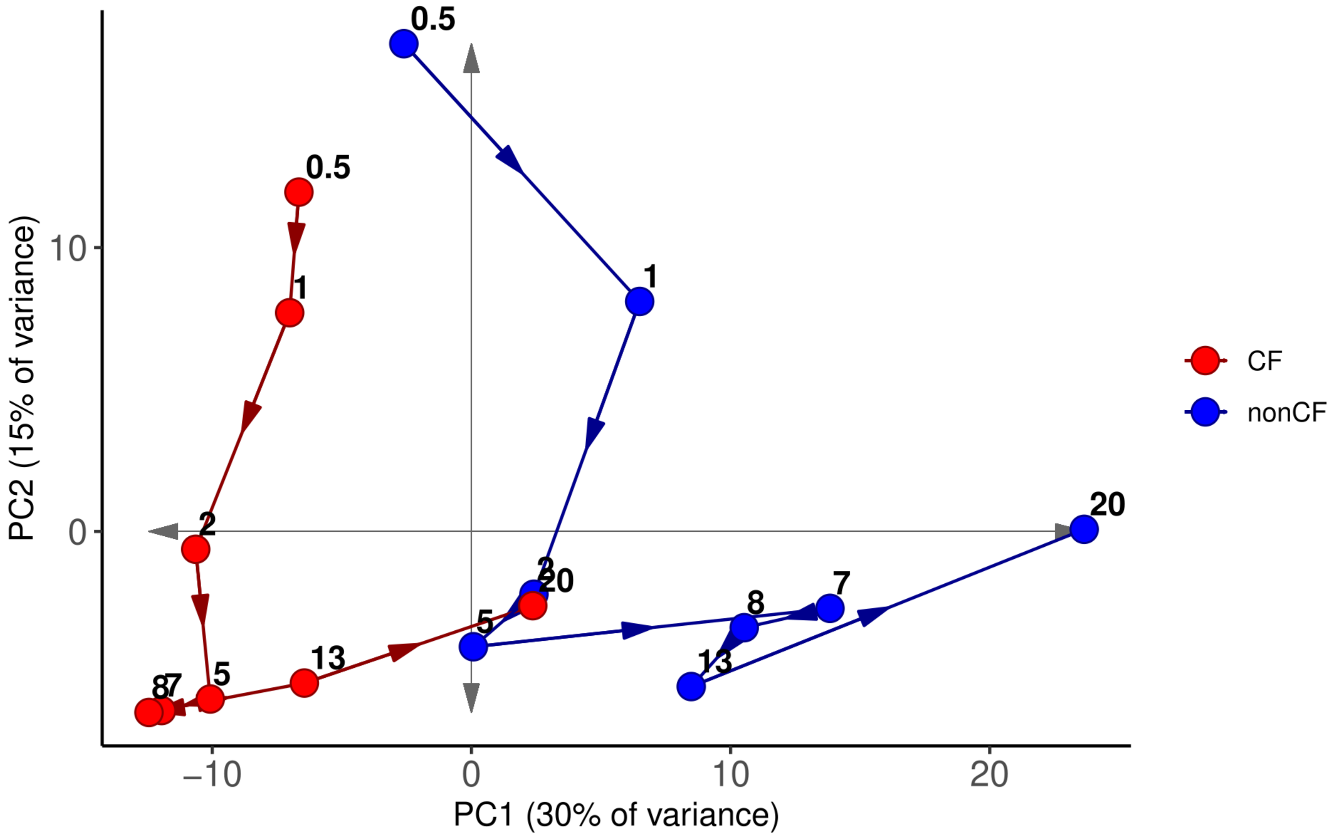

Figure 11S: RNA expression of the indicated genes in newborn liver. The species of measurement is indicated. Each dot represents measurement in an independent sample. * FDR<0.05.

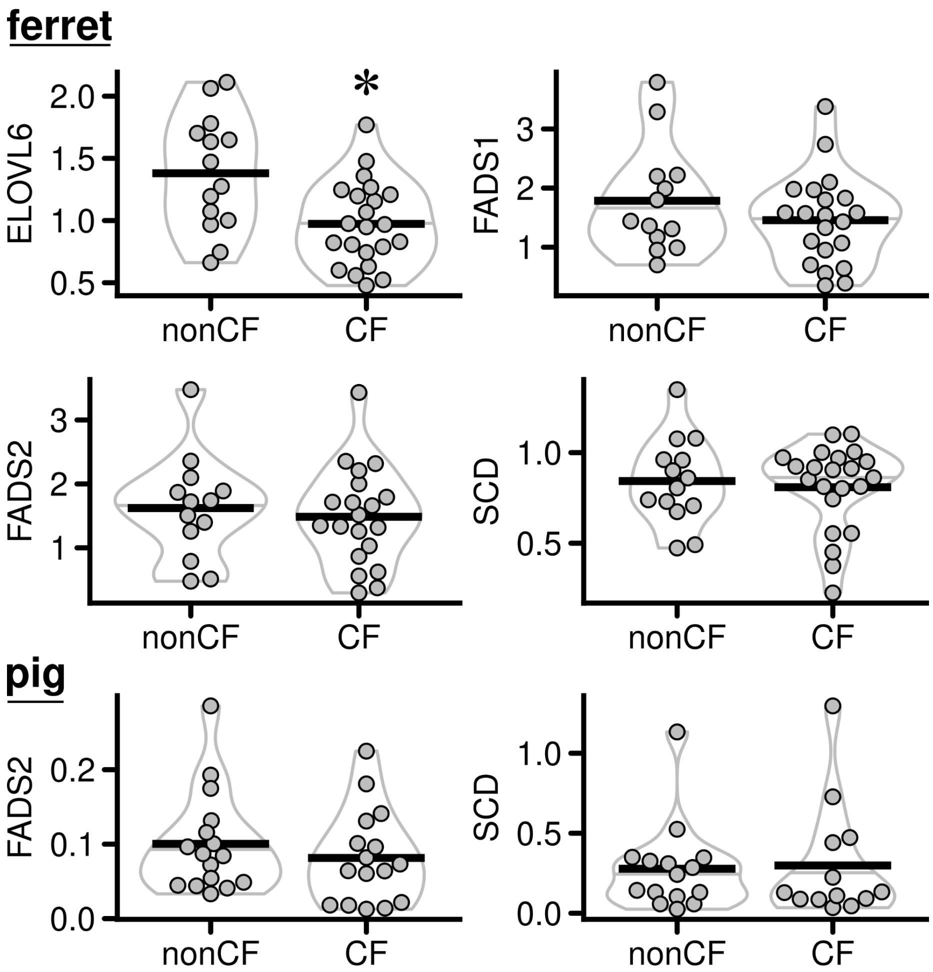

Supplement: Supplementary Figures S1-S11 and Tables S1-S4 [file CS-2022-0450_supp.pdf]
